# On the Mechanical Performance of an L-PBF 316l Part Using the Performance-Line Instrumented Indentation Test (PL-IIT)

**DOI:** 10.3390/ma18071462

**Published:** 2025-03-25

**Authors:** Giovanni Maizza, Faisal Hafeez, Alessandra Varone, Roberto Montanari

**Affiliations:** 1 Department of Applied Science and Technology, Politecnico di Torino, 10129 Torino, Italy; faisal.hafeez@polito.it; 2 Department of Industrial Engineering, University of Rome “Tor Vergata”, 00133 Rome, Italy; alessandra.varone@uniroma2.it (A.V.); roberto.montanari@uniroma2.it (R.M.)

**Keywords:** nanoindentation, L-PBF, 316L stainless steel, performance line instrumented indentation test (PL-IIT), ISE-free property, loading stiffness rate

## Abstract

While L-PBF research continuously expands technologically towards more complex-shaped components and effective scanning strategies, the customization of the mechanical performance of these components to specific applications is still challenging. The presence of high process-induced residual stress levels frequently makes the current (standard) mechanical testing procedures ineffective or even inappropriate. The current engineering design principles cannot be applied to L-PBF components as the available mechanical properties are apparent (i.e., space and residual stress dependent properties). It is the aim of this work to overcome the aforementioned limitations by presenting a comprehensive methodology that can be used to determine the mechanical performance of an L-PBF 316L deposit along (five) pre-specified directions, denoted as performance lines (PLs), and in six special key regions, denoted as performance zones (PZs), through the nanoindentation test (PL-nIIT). The PLs determine the gradients of the indentation properties across the deposit, while the PZs exhibit the orientation-dependent mechanical performance in a specified number of regions of the deposit. The latter can be used for benchmarking, mechanical design, or performance customization. The frequently resorted to indentation modulus and hardness have thus been complemented with a new indentation size effect-free property (i.e., the loading stiffness rate, LSR) to help discriminate the presence of residual stress at different depths in the given deposit. A decreasing mild compressive residual stress was determined along the build direction of the deposit as revealed by the decreasing values of the relative LSR, H_IT_, and E_IT_ (from the root to the top dome, i.e., 47.8 to 43.4, 2.57 to 2.49, and 216 to 202 GPa, respectively).

## 1. Introduction

Laser beam powder bed fusion (L-PBF), which is also known as selective laser melting (SLM), offers an extraordinary shaping versatility and fine process control to the fabrication of components that have an intricate geometry. Its directional energy input can in fact impart unique non-equilibrium, layer-by-layer, heterogeneous microstructures with a possible macrotexture that is connected to outstanding anisotropic properties. However, L-PBF microstructures can sometimes embed certain undesirable fabrication defects, such as unmolten powder, poor adhesion, porosity, and residual stresses [1,2]. Such L-PBF microstructures build up because of the intimate interplay between various external process factors (e.g., fabrication parameters, laser parameters, and baseplate features) and complex internal factors (e.g., heating rate, thermal gradient, and cooling rate). The latter are in fact responsible for the intricate coupling that takes place between locally repeated non-equilibrium metallurgical phenomena (e.g., melting, solidification, precipitation/dissolution, recrystallization, etc.) during layer-by-layer fabrication [3,4,5] and for the inherent build-up of residual stresses. Thus, detailed investigations on a thus formed microstructure, together with its anisotropic mechanical properties, and any eventual residual stresses are essential to optimally tailor the mechanical performances of L-PBF parts [4,6] to satisfy the requirements of specific engineering applications.

Several studies have focused on the L-PBF of the SS 316L alloy, because of its outstanding combination of chemical and structural properties, which are especially beneficial for the aerospace, automotive, biomedical, marine, chemical, and power-plant industries. Moreover, the vast number of articles that have been published on this alloy also confirms its choice as a benchmark for L-PBF alloys. The main research challenges currently being faced by the L-PBF of the SS 316L alloy concern the lack of a systematic local correlation between the complex heterogeneous/anisotropic microstructure [6,7] and the mechanical properties [8], as well as their inherent optimization through the optimal tuning of the process parameters. A related research challenge refers to the setting up of appropriate non-destructive or quasi-destructive testing methods to assess the local properties (or global performances) of both samples and integral components. Microstructural anisotropy may result from a variation in the grains (of different size and/or shape) growing along different planes [9] or preferred growth direction, as driven by a thermal gradient [10]. Grain anisotropy has recently been accounted for by means of 3D electron back scatter diffraction (3D-EBSD) techniques [11]. A successful reduction in microstructural anisotropy has recently been reported as a result of promoting recrystallization [6,9], although some weak crystallographic components along the build direction. Zhongji Sun et al. [9] succeeded in encouraging grain growth in the <011> direction of an L-PBF 316L deposit, rather than in the typical <001> direction, by varying the laser power and scanning strategy. Moreover, they were able to enhance the tensile strength (~16%) and ductility (~40%) of the sample. Uddin et al. and Kurdi et al. [12,13], after investigating the effects of laser volumetric energy density (VED) on the anisotropic indentation properties (E_IT_ and H_IT_), found that when the VED was increased, the grain size became larger, wider, less elongated, and more oriented along the <101> direction. Reference [14] conducted research on the influence of the scanning strategy on the formation of the microstructure and texture.

The measurement of residual stress (RS) in L-PBF deposits is currently hindered by a lack of industrially viable, non-destructive methods that can be used for complex components. Indeed, RS, which tends to self-equilibrate in a complex manner across a volume, because of repeated thermally induced metallurgical processes (remelting, solidification, tempering, recrystallization, precipitation/dissolution, etc.) of the layers underneath, is frequent in L-PBF deposits [15,16]. Although compressive RS may enhance the fatigue life, tensile RS is generally more detrimental as it may accentuate corrosion, lead to a drop in the mechanical properties or cause cracks in the final component. The unique complexity of the microstructure and mechanical response of an L-PBF 316L deposit has recently been reported [8] through the use of nano- and macro-scale instrumented indentation tests (IIT). Although macro-IIT is able to sense the tensile-test-like mechanical response of a deposit, which may involve a volume of several grains that embody defects, grain boundaries (GBs), and precipitates, it is very sensitive to long-range RS, especially in sliced L-PBF samples [8] in which the initial RS state has been altered. When measuring the macroscale indentation properties (at 600 N) in an L-PBF 316L sample, the unbalanced tensile-RS, caused by the slicing of the sample, resulted in (i) a notable crack of the residual imprint, and (ii) abnormal indentation curves, after unloading, caused by the concomitant partial relaxation of the tensile-RS and a loss of compliance between the indented sample and its holder [8]. Moreover, the measured indentation moduli were suspiciously low. On the other hand, the nano-IIT (≤10 mN) proved to be affected less by RS, as a result of the shallower indents [8]. Interestingly, other authors have employed nano-IIT to sense anisotropic indentation properties as a function of the crystal orientation of the grains in an L-PBF SS 316L alloy by varying the laser volumetric energy densities (VED) [12,13].

However, nano-IIT suffers from an important shortcoming, that is, the indentation size effect (ISE), which becomes crucial when it is necessary to compare indentation hardness for different peak loads. ISE causes higher indentation hardness values at lower penetration depths [17] and becomes negligible at larger penetration depths than 100 nm [18,19]. However, a tip blunting effect may also come into play at these depths, that is, an effect which requires an ad hoc calibration to find the correct area function of the indenter [20]. Thus, ISE-free indentation properties are of paramount importance to sense L-PBF parts at different depths (or peak loads) in an accurate manner. Several authors have searched for a better fitting of the measured indentation curves in attempts to obtain a better understanding of the ISE. Some of them for instance [21,22] used Bernhardt’s law rather than the common power law, and they in fact achieved a more accurate fitting of the indentation curves (ICs) and obtained a new indentation parameter, as an output, from the loading curve, which was largely load (or depth) independent. The standard Oliver and Phar method [23] instead suggests extracting the indentation properties from an unloading IC via a power law fitting function.

In this work, we have fitted the measured loading ICs with Bernhardt’s law to ensure accurate and universal (i.e., load-independent or depth-independent) nano-indentation properties. This law allows a pure ISE-free indentation property to be estimated from the loading curve, which can be compared with the standard indentation properties obtained with the [23] method.

At present the engineering design of efficient structural L-PBF components is strongly limited by the lack of standard procedures enabling their mechanical qualification. The current major limiting factor is related with the tensile test measurement of space dependent material properties which naturally couple with short- and long-range residual stresses originating from the fabrication process. To date, engineering design of components is mainly based on tensile tests which, in the case of L-PBF components, provides bulk ‘apparent’ properties. However, for L-PBF components two obstacles currently hinder the regular engineering design or verification process: (a) the lack of criteria suggesting the identification of the key region(s) of the component to be mechanically assessed; (b) the lack of mechanical design properties applicable to the key region. As the properties across the L-PBF components change pointwise these should more correctly be denoted as performances. This, in turn, imposes a new methodological approach to mechanically test, design, verify, and even fabricate AM components. As tensile test cannot provide the mechanical performances at the required key regions of the L-PBF component, local testing methods, such as instrumented indentation test, become more appropriate. However, the available international code on instrumented nanoindentation test [24,25] cannot apply to sample materials affected by residual stresses, hence alternate approaches are needed to overcome such current limitation. The next section presents a convenient nanoindentation strategy that allows the local mechanical performances of the deposit to be determined in arbitrary structural AM products.

## 2. Materials and Methods

### 2.1. The L-PBF of 316L Build

**Figure 1:**
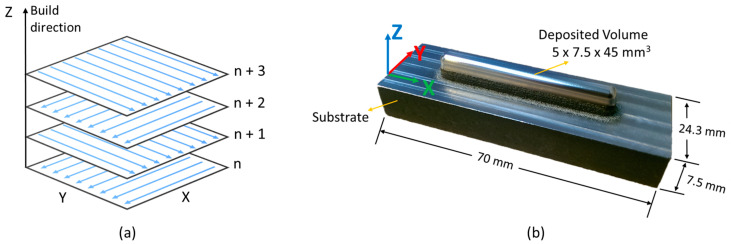
(**a**) Scheme of the scanning strategy used in the deposition process and the axis reference frame. (**b**) As-deposited build block (AISI 1020 steel substrate) after top-surface finishing.

An alternating X- and Y-scan strategy (with a 90° interlayer angle) was adopted to deposit AISI 316L steel (American Iron and Steel Institute, Washington, DC, USA) on a base plate made up of AISI 1020 steel (Figure 1a). The final deposit measured 5 × 7.5 × 45 mm^3^ and it was deposited on a 24.3 × 7.5 × 70 mm^3^ base plate (Figure 1b).

The feedstock consisted of a pre-alloyed powder (LPW-316-AABE) with an average grain size of 44–88 µm; the chemical composition (in weight percentages) of the final deposit is given in Table 1. The *Z*-axis corresponds to the building direction. The deposition was carried out with a laser power of 350 W and a scan rate of 300 mm/min. The dome (top head) surface of the build block was finished off by grinding. The alternating scanning strategy added a particular heterogeneity to the microstructure, which consisted of a periodic microstructure composed of two repeated distinct layers (stripes). As the overlying layer melted the one beneath, partially recrystallized grains formed in the remelted interlayer zone [26].

### 2.2. Mechanical Performance of AM Products

The mechanical performance of AM components is hereafter assigned to specific key regions that are relevant for the mechanical design of the components because they are directly linked to one or more system parameters related to the fabrication process. If the mechanical response is known in such regions, both the local and global performances of an industrial AM product can be virtually optimized or benchmarked as a function of the system parameters. These system parameters include the materials (deposit and substrate), geometries (deposit and substrate), the laser parameters (power, scanning speed, spot radius, etc.) and fabrication parameters (layer thickness, hatch distance, and scanning strategy), as well as physical parameters (pre-heating temperature of the substrate and powder, auxiliary cooling systems, ambient temperature, etc.).

**Figure 2:**
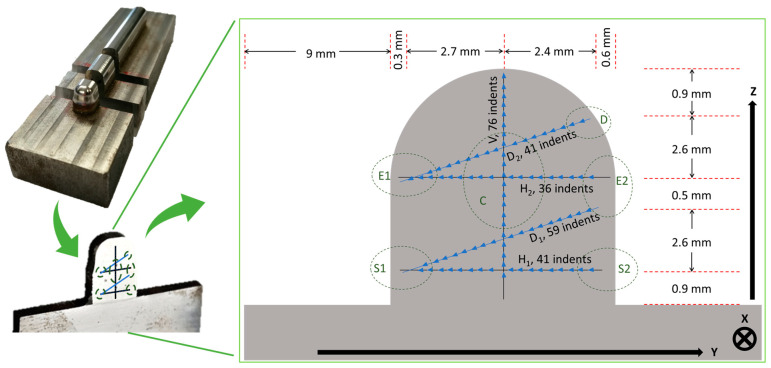
Sketch of the nanoindentations over the surface of the deposit (Y-Z plane) of the sample; definition of the five performance lines (H1, H2, D1, D2, V) and the six performance zones (S1, S2, E1, H2, D, CZ or C). The X-scan and Y-scan layers are on average 400 and 380 μm thick; the H1 line runs along the centerline of the 3rd Y-scan layer; the H2 line runs along the centerline of the 8th X-scan layer. The insets show the sample extracted from the original L-PBF 316L deposit.

For simplicity, we have here illustrated the proposed methodology for the case of a sliced sample extracted from an original L-PBF SS 316L build, which is shown in Figure 1. In general, when extensive regions of an AM product are sensed by means of nanoindentation arrays or maps, their mechanical response cannot be reduced to one single average value of each indentation property. Indeed, all the indentations in that region contribute to the definition of its local mechanical response. In this work, we selected some specifically orientated indentation lines that crossed the tested surface in such a way that, after appropriate post-processing, they closely reflected the mechanical response. This concept is hereafter used to introduce the nominated performance lines (PLs) along which the nanoindentation arrays were carried out. Considering the selected scanning strategy for the given L-PBF SS 316L build, five performance lines (see Figure 2) were designed over the Y-Z plane of the sample. While the horizontal and vertical lines traced the mechanical response and any anisotropic effect along the coordinated axis (X and Y), that is, along the horizontal stripes and across all layers of the sample, respectively, the inclined lines traced any cross-anisotropic effect from bottom to top and edge-to-edge. The lower horizontal line were located at the bottom of the deposit to sense the close influence of the substrate. The mid-height horizontal line was designed to mainly sense the lateral edge effects and, in a mitigated manner, those from the substrate and the dome. The axial vertical line was intended to trace the mechanical response by mitigating the effects of the lateral edges but properly accounting for those at the substrate and the dome. The intersection of the PLs with the edges of the deposit identified the so-called performance zones (PZs). The size of a PZ was determined by a pre-specified number of indentations lying along the PLs. Given the symmetry of the sample, all the PLs were divided into three smaller segments of equal length, and an equal number of indentations were considered. As the indentation modulus was found to suffer more than other properties from large deviations, the maximum values of the indentation properties extracted from each segment were assigned to represent the local mechanical response in each PZ. Such large deviations were ascribed to grain anisotropy and residual stresses and denoted hereafter as heterogeneities. Symmetrical considerations suggested that the core zone (CZ) was special in that it can mitigate all system influencing factors, and thus, can be regarded as the most representative zone of the L-PBF 316L sample.

### 2.3. Microstructure

The metallographic inspection and nanoindentation measurement samples were sliced, starting from the center of the block, with the cross-section perpendicular to the X-direction. The nIITs were performed on the Y-Z plane, with the loading direction along the X-scan direction. Sample flatness was ensured by mounting the sample onto resin, milling and grinding it with 300 to 4000 grit SiC smearing papers and finally mirror-polishing it with 6-, 3-, and 1 µm particle size diamond suspensions. The polished samples were etched for 65–75 s with aqua regia and then inspected through the use of light microscopy (DMI 3000 M, Leica, Wetzlar, Germany) and scanning electron microscopy coupled with energy dispersive X-ray spectroscopy (SEM—FEI M-EDX Sirion 400 NC, Thermo Fisher Scientific, Hillsboro, OR, USA). Electron backscatter diffraction (EBSD) analysis (TESCANS 9000G, Tescan group, Brno, The Czech Republic) was carried out at a 1.5 kX to 12 kX magnification and 20 kV. The L-PBF SS 316L alloy microstructure did not show any significant porosity or other processing defects.

### 2.4. Loading Stiffness Rate (LSR)

It is well known that the loading segment of the P-h curve of indentations with sharp indenters can be described with a parabola (i.e., $P=Ch^{2}$) for a perfect elastoplastic material [27], where P and h are the load and indentation depth, respectively. The proportionality constant C is a load-independent geometrical factor that accounts for indentation compliance [21,28]. Although Kick’s law has been utilized in many studies to fit experimental ICs, considerable deviations have frequently been observed, especially at their origin. Bernhardt [29] proposed a two-term equation to mitigate the shortcomings of Kick’s law:

(1) $$P=bh+ah^{2}$$

The above two-parameter (b and a) law permits one to accurately describe the deviation of the experimental data from the ideal parabolic law, which is frequently linear in-depth h, as confirmed by the vast number of materials that fulfill Bernhardt’s law (Equation (1)). Bernhardt’s law can alternatively be expressed, for post-processing ease purposes, in terms of the secant stiffness, S_h_, defined as

(2) $$S_{h}=\frac{P}{h}=b+ah$$

where the subscript *h* specifies penetration depth h. Equation (2) has been utilized by many authors to prove different arguments or theories. In this work, the secant stiffness was introduced with a different purpose from those prescribed in the past. It is in fact different from its loading tangent stiffness counterpart as presented in the definition of Martens’ hardness. It should be noted that both stiffness parameters can be simply related to each other in the (frequent) case where the measured indentation curves can be fitted with a power law. In this case, the power exponent is the proportionality factor which links the secant stiffness and the tangent stiffness. The secant stiffness enabled us to compute the stiffness of the material as a simple load to depth ratio (Equation (2)), rather than facing inaccurate derivatives. In place of fitting the conventional indentation loading curve, a simple line regression was needed to fit the S_h_ vs. h curve. The outcome was a pair of characteristic parameters, the “a” slope and the “b” y-intercept. Previous studies proved that many metallic and ceramic materials could be described by a straight line over a rather ample depth range of h_max_, except near the origin (due to ISE, indenter blunting and other surface anomalies). As the indentation properties are typically defined near h_max_ (or F_max_), we defined the depth range (h_max_-h_s_) over which the secant stiffness was truly linear as (h_max_-h_s_)/h_max_ = ∆h and the associated Bernhardt’s parameters as b_∆h_ and a_∆h_. The latter is generally denoted as the loading stiffness rate (LSR), and it is frequently invoked in the next sections. It should be noted that the wider ∆h is, the closer the LSR is to a material property.

### 2.5. Performance Line Nanoindentation (PL-nIIT)

Although nanoindentation mapping is becoming increasingly popular, we believe it is highly inefficient when applied to an industrial context. A single nanoindentation accounts for a small fraction of the desired macroscopic response of a representative macro-region of the deposit that may span several hundreds of microns, or even more. A suitable criterion is thus needed to convert a nanoscopic mechanical response from nanoindentation to a local macro-mechanical response of a broader region of the deposit. In general, the macro-mechanical response of an AM product is determined by its microstructure and, eventually, by its internal defects, RS, and texture (if present). These product features are controlled by three internal (process) factors, that is, the thermal gradient, the cooling rate, and the solidification rate. These, in turn, arise from a multitude of external processing factors (e.g., the laser parameters, convection cooling, the scanning strategy, the substrate temperature, the dilution ratio at the interface between the substrate and the deposit, etc.). A pre-specified number of performance zones (PZs) can be defined along the periphery of the deposit to design its optimal geometry, microstructure, residual stress state, and anisotropy as a function of one or more external processing factors by monitoring the internal process parameters. As these boundary regions directly impact the local macro-mechanical performance of the deposit, they are denoted as performance zones. An appropriate connection among these PZs, obtained by means of nanoindentation, can explain the process-driven criterion of the searched for nanoindentation scheme based on the performance lines (PLs). The considered L-PBF 316L deposit consisted of five PLs, that is, two horizontal ones (H1, H2), one vertical one (V), two 40° inclined diagonal lines (D1, D2) and six PZs (D, S1, S2, E1, E2, CZ) (see Figure 2). Accordingly, the resulting nanoindentation scheme was denoted as 5PL-nIIT. Each PL was partitioned into three segments of equal length and number of indents to convert the array of lineal nanoindentation properties into local macro-mechanical responses at the PZs. The length of the segments delimited the extension of the PZs, while the indentation properties over each segment contributed to determining the mechanical response of each PZ. Each PZ was designed to capture one or at most two distinct external process factors. A special PZ of the deposit was the one that resulted from the interception of the internal segments across its core, which was denoted as the core zone (CZ). This zone, owing to the symmetry of the investigated system, was defined as a reference zone as it was influenced by all aforesaid system factors albeit in a mitigated manner. Thus, it can be used for benchmarking the mechanical response of the deposit under different system influencing factors. The top surface of the dome acted as a distinct convection cooling factor, which was here accounted for by the D zone. In this study, the D zone did not play a relevant role, compared to the lateral edges (E1 and E2) of the deposit (Figure 2). Any significant difference in mechanical response between E1 and E2 could, in addition to the cooling effect from the lateral edges, help account for any deviations of the mechanical properties (i.e., heterogeneities or anomalies) along a stripe, with a reduced influence of the substrate. Indeed, the latter generally plays a major role, especially in the early stages of the deposition process, via its appreciable heat sink effect and its dilution ratio with the initial layers. The conduction cooling effect along the deposit–substrate interface was mainly captured in the S1 and S2 zones, although useful information could also have been collected across the central zone along the H1 line. We did not specify the latter as PZ, although its indentation properties across this zone were available since no additional effects were expected in this zone which had not been accounted for by both S1 and S2. The horizontal H1 and H2 lines were designed to track the microstructure gradient along either the X- or Y-scan deposited layer. As the H1 line ran closely along the substrate and H2 ran parallel to but at a distance from the substrate, both could have been used to discern the overall impact of the latter along either stripe, including the effects of dilution (primary heterogeneity), the heat sink and any residual stress/strain effects. These external factors are known to have a marked impact on the global mechanical performance of AM products. The V line axially crossed the build sample from the bottom (substrate) to the top (dome), and it could therefore have been used to assess the main multilayer structure gradient, as well as the combined heterogeneities and RS. The intersection between the V and H2 lines defined the CZ of the deposit and determined its representative mechanical response. The inclined lines (D1, D2) were effectively used to track the changes in the microstructure over several X- and Y-scan layers, and they could therefore be used to detect the re-solidification and tempering effects due to the overlying layers. Thus, the PZs, with their individual mechanical performance, offered a useful means of optimizing the deposit design and the deposition conditions or of benchmarking its performance as a function of the external process parameters.

As the gradients of the measured indentation properties across a deposit (i.e., along the PLs) can deteriorate because of excessive fluctuations, a suitable approach should be undertaken to make them as smooth as possible. Among the various smoothing and averaging methods investigated in this work, the best results were obtained for the averaging method given by Equation (3), which is inspired by the definition of average normal anisotropy [30], widely employed in sheet-forming operations. Here, it was employed to smooth out the effect of the outliers, in place of simply neglecting them, to specifically determine eventual gradients in the indentation properties. For example, for the case of the indentation hardness measured along a generic PL, we obtain

(3) $$mean_{H_{IT}}=\frac{{H_{IT}}_{i-1}+{2\times H_{IT}}_{i}+{H_{IT}}_{i+1}}{4}$$

This equation can be experimentally justified by considering two general observations: (a) local indentation properties might not always result in the real local microstructure features being sensed, and instead may combine anomalies which inevitably deteriorate the searched for trend; and (b) dramatic changes are not expected between two adjacent indentations of any indentation property, except for the undesired presence of accidental defects, pores, precipitates, etc. In such a case, such abnormal properties can safely be discarded when a general trend needs to be estimated. Indeed, the indentation modulus is generally more sensitive to anomalies than indentation hardness. Thus, this formula attempts to filter out such anomalies by weighing their local contribution (Factor 2) against those (if any) from the neighborhood (Factor 1).

We defined the local mechanical response in the PZs, with respect to any indentation property, by considering the maximum value of any property over each pertinent segment. This criterion prevents the unfortunate event of large dispersions in the indentation properties impeding a net comparison among the mechanical responses from the PZs (Figure 2). Finally, as the local response of PZs is generally expressed in terms of indentation properties extracted from the pertinent segments, their specific orientation may be considered to provide local information on the microstructure, heterogeneity, and RS gradients of any PZ.

### 2.6. Indentation Test Conditions

The nano-indented area was reduced according to Figure 2 to prevent any unwanted edge effects. The arrows in Figure 2 indicate the progress of the indentations (i.e., toward the left or toward the top).

Nanoindentations were conducted using a Hit 300 device (Anton Paar GmbH, Graz, Austria), equipped with a Berkovich diamond tip. The surface of the indented sample was previously polished to minimize surface heterogeneities. The indentation cycle (peak load, holding time, and loading and unloading rates) was fixed throughout the experiment. Prior to the measurements, the area function was pre-calibrated according to the [24] standard to account for the frame compliance of the test device. Any possible thermal drift effect was minimized by keeping the sample within its holder for 24 h [31]. The separation distance between two consecutive indents was set to 80 μm. The H1, H2, D1, D2, and V lines (Figure 2) were filled with 41, 36, 59, 41, and 76 indentations, respectively. Since the grains of the L-PBF 316L alloy were relatively large, in comparison to the indent size (4 µm pyramid height and 1.4 µm depth), the indentation properties were barely influenced by the grain boundaries. Indeed, relatively coarse grains are likely to act as single crystals in the nIIT. Preliminary indentation tests with different peak loads (10, 100, and 150 mN) were carried out to exclude any evidence of ISE. As a result, 100 mN was selected as the maximum peak load for a 10 s dwell time, while 0.5 mN/s was chosen for the loading and unloading rates.

The quantitative correlation obtained via the Pearson correlation coefficient [32] between the microstructure and the averages of the indentation properties (H_IT_, E_IT_) along the PLs, was strengthened by involving new standard indentation properties (e.g., the total work, W_t_, the unloading work, W_u_, the plastic work W_p_) and parameters (e.g., the maximum penetration depth, h_max_) [24,25]. Pearson’s correlation coefficients, which were equal to +1, indicated a close direct correlation between two compared quantities; conversely, the −1 values indicated a close indirect correlation. Because of the large deviation of the indentation modulus values, the regular H_IT_ and E_IT_ values were complemented with a new indentation property, LSR, whose significance is here explained by the fact that the indentation data plotted in the S_h_ vs. h plane fit well with a straight line (with correlation factor closer to 1) over a significant depth range. Under this condition, the line slope (LSR) reliably represents the true elastoplastic response, under the selected indentation peak load, except for a small range close to the origin. The specification of either the load range or the depth range is important in the presence of an RS state, since this is closely linked to the microstructure properties, whose degree and distribution may change along the depth. Moreover, it should be recalled that LSR is typically extracted upon loading, without any prior knowledge of the contact area. Conversely, H_IT_ is extracted at the maximum peak load (upon incipient unloading) with a compliance enforced measurement of the contact area based on the calibration procedure. Furthermore, any attempt to cross link the indentation properties, H_IT_, E_IT_, and LSR could be hindered by the presence of heterogeneities, anisotropy, and residual stresses along the PLs and in the PZs of the investigated L-PBF 316L build sample. The standard deviation (SD) of each measured indentation property offered a useful statistical spread index of the respective averages in absolute terms. Conversely, the relative standard deviation (RSD = SD/mean) index can provide a more effective index to discern the influence of microstructure heterogeneities, defects, and artifacts on each indentation property.

## 3. Results

### 3.1. Microstructure of the Deposit

**Figure 3:**
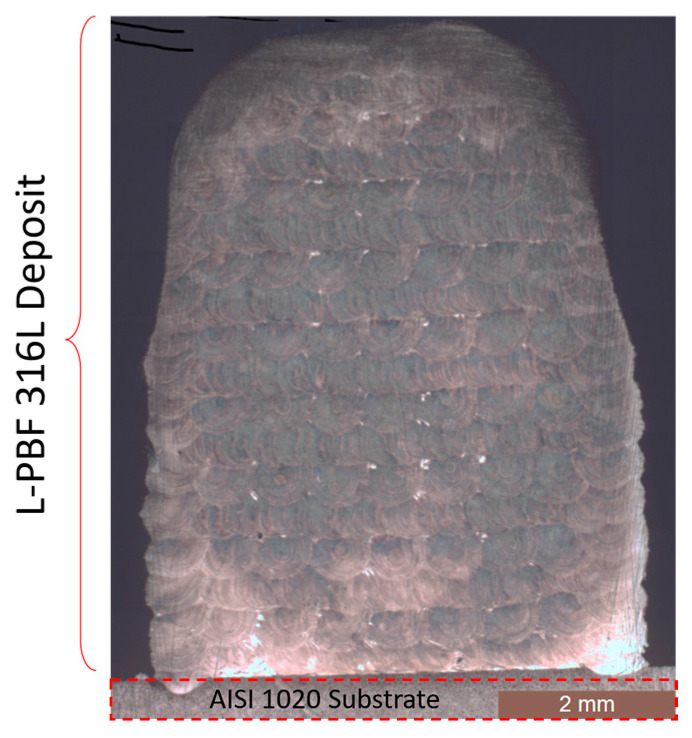
Optical micrograph of the 316L deposit (18 alternating X- and Y-scan layers) on AISI 1020 steel substrate.

**Figure 4:**
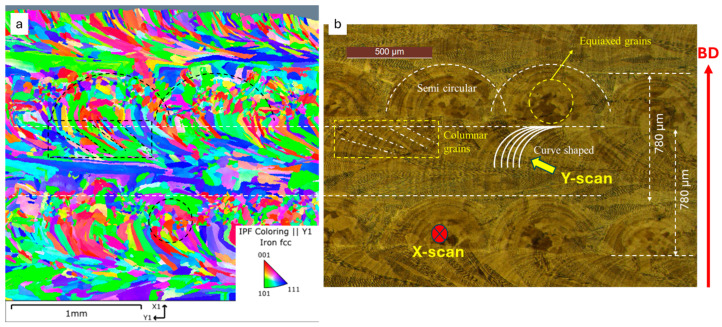
Distribution of the grain orientations in the Y-Z plane from two distinct (X- and Y-scan) stripes of the L-PBF 316L build sample showing characteristic columnar and equiaxed grains: (**a**) inverse pole figure (IPF) (**b**) OM micrograph (upside down view to match the microstructural features with the EBSD figure).

**Figure 5:**
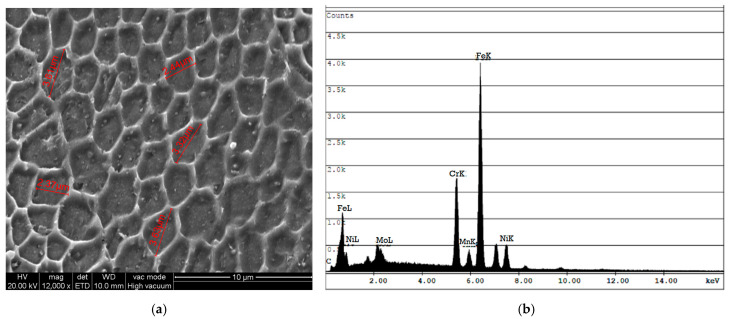
(**a**) Typical cellular structure observed through the SEM microscope comprising equiaxed cells; (**b**) EDX spectra detected in a small region of the microstructure shown in (**a**).

The deposit consisted of 18 alternate X-scan and Y-scan (90° rotation) layers, the first one that was adjacent to the substrate was a Y-scan layer, while the last one was an X-scan layer that contoured the curved dome tip (see Figure 3). The entire deposit was completed with contour (X 7-scan) passes along its edges to refine the surface finish. The microstructure of the polished and etched sample was inspected by means of EBSD (inverse pole, Figure 4a) and its core was observed by means of optical microscopy (Figure 4b). Consistent with the results of another work [33], no significant porosity was detected. Figure 4a shows the crystallographic orientation of the grains on the Y-Z plane. The 90° alternating scan strategy promoted a combined cellular and columnar substructure, which is shown in Figure 5a, obtained via scanning electron microscopy (SEM) at a 1.5 k–12 k magnification. Figure 5b shows an energy-dispersive X-ray (EDX) spectrum of the deposit, taken at the same location as the SEM measurement, which resembles the chemical composition of the powder shown in Table 1. Figure 4b shows the typical semi-circular (X-scan) and curved (Y-scan) shapes of the solidified weld pools. Unlike the curved shaped stripes, which generally include elongated grains with a cellular substructure, the semi-circular stripes mainly consist of large equiaxed grains, although they may occasionally involve columnar grains of an epitaxial nature. This layout was also observed by other investigators [34,35,36]. The two alternating scans tended to break down any possible long epitaxial grains that crossed multiple stripes, thereby reducing the macro-anisotropy of the sample. The SEM results indicated that the cellular structure of the grains was mostly equiaxed at the center of the solidified melt pool, and it was elongated at its boundary [37]. Figure 4b indicates the presence of melt-back phenomena [38], as a consequence of the partial re-solidification of alternating layers, which led to the formation of relatively fine equiaxed grains. The curved stripes generally exhibit minor micro-elemental segregation at the melt front (Figure 4b). Overall, the observed microstructure morphologies are consistent with those reported by other researchers on the L-PBF of SS 316L e.g., [39]. Figure 4a also reveals the presence of minute recrystallized grains in the X-scan/Y-scan interlayer regions that could have originated from the combination of the rapid thermal cycling and intense plastic deformation caused by the local differential expansion/contraction between the two layers [26].

### 3.2. Crystallographic Texture

The grains that formed during the deposition in the X-scan direction were mostly oriented along directions somewhere between the <001> (red) and <101> (green) of the main crystallographic orientations, and they showed a minor dependence on the <111> (blue) orientation, as also reported by [40,41]. Conversely, the grains deposited along the Y-scan direction were mostly oriented toward the <111> (blue) directions. We observed that the orientation of the latter grains was frequently altered after the deposition of the overlying layer, thereby mitigating the presence of any initial fiber texture. Additionally, a minor fraction of the deposited grains was oriented toward such intermediate orientations as the <112> (violet), <102> (yellow) and <212> (cyan) directions. Indeed, Figure 4a shows recrystallized grains at the interlayer regions. We can confirm that the alternating X- and Y-scan deposition strategies promoted a multi-component crystallographic direction [42]. Moreover, according to Uddin et al. [12], the <111> grains were expected to be the stiffest grains and the <001> grains the most compliant ones. However, we did not expect a pronounced anisotropic response from even the strongly oriented grains from equilateral Berkovich indentations, such as the ones that were analyzed here, especially whenever the elastoplastic influence of the surrounding grains was also accounted for. Thus, a mediated 3D elastoplastic behavior, with a certain micro-texture effect (if present), was expected from the nanoindentation responses across the grains of the investigated L-PBF 316L alloy.

### 3.3. Indentation Properties

**Figure 6:**
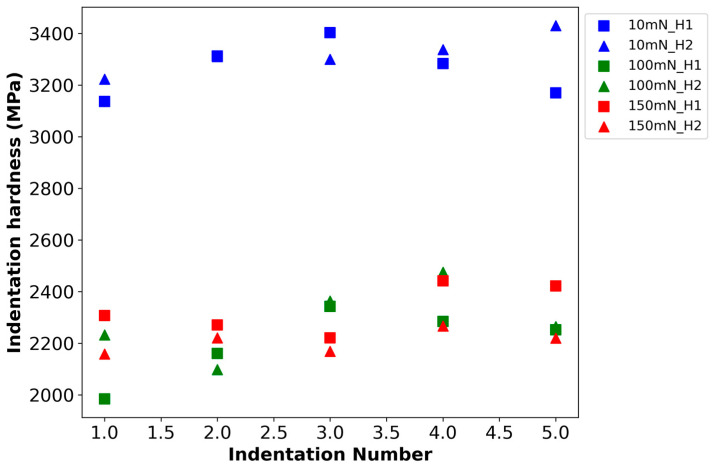
Assessment of the indentations size effect (ISE), via indentation hardness measurements, along the H1 and H2 lines for three peak loads.

Figure 6 shows the results of the ISE assessments along the H1 and H2 lines considering five numbers of indentations repeated at three different maximum loads (10, 100, and 150 mN). ISE was clearly prominent for 10 mN where H_IT_ ranging to 3.29 ± 0.08 but did not influence high loads, i.e., 2.24 ± 0.12 at 100 mN and 2.27 ± 0.06 at 150 mN peak loads. However, there was less spread in the indentation hardness values at 10 mN and 150 mN and more at 100 mN.

**Figure 7:**
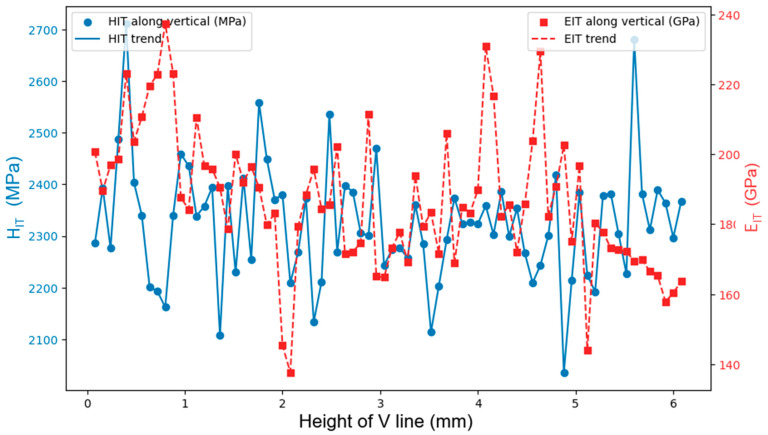
H_IT_ and E_IT_ values along the V line (with X axis showing the distance or height of the V indentation line, each point is the indentation site, with an 80 µm inter-separation distance between two indents).

Figure 7 shows an exemplary scatter plot of H_IT_ (blue filled circles) and E_IT_ (red filled squares), along the V vertical line, versus the indentation locations (76 in total). The arrows in Figure 2 depict the convention used for the PL orientation applied in Figure 7. It shows difficulty we encountered in finding a discernible trend for H_IT_ and E_IT_ along the V line (and other PLs) or any correlation between these properties and, hence, recourse was made into more advanced means, such as the Pearson correlation factor approach. Handling of the outlier required further investigation. It was found that very few of them were unphysical whereas other ones were ascribed to special, though unknown states of the material and thus were included in the subsequent post-processing. It will be seen as proper averaging of the data, including unusual peaks, helped discern the searched gradients of the indentation performances across the deposit.

Table 2 lists the mean values and %RSDs of various indentation indices from the five PLs, that is, H1 (41 indents), D1 (59 indents), H2 (36 indents), D2 (41 indents), and V (76 indents). The reported %RSD value for H_IT_ was nearly constant, but it noticeably varied for E_IT_, with H2 and V lines, showing the highest dispersion of such properties. The small variation in %RSD in H_IT_ followed that of h_max_. Hence, it could not be of considerable help in the discrimination of the local mechanical responses in the PZs. The loading segment of the ICs was fitted with a power law which parameters (the strength coefficient and the power exponent) were given by K_L_ and n_L,_ respectively. Worthy of notice is that the %RSD values of K_L_ were surprisingly quite large meaning that the loading curves markedly deviate from the expected pure elastoplastic behavior across the investigated depth ranges. This was also confirmed by the n_L_ values lower than two, theoretically expected for pointed indenters. It was found that the characteristic depth range, Δh, of the L-PBF 316L alloy was about 85% for nearly all the indentations along the PLs, hence defining the a_85_ and b_85_ fitting quantities for the investigated materials. These two parameters along with the h_max_ and the %RSD were discussed more thoroughly in Section 4.

Table 3 shows the degree of correlation between the mean values of the most relevant PL indentation indices, as obtained by means of Pearson’s correlation coefficient. It emerges that H_IT_ is strongly but inversely correlated with h_max_, but h_max_ is directly correlated with W_p_ and W_t_. Conversely, the latter is inversely but only slightly correlated with a_85_. As expected, H_IT_ is also inversely correlated with the total work (W_t_) and the plastic work (W_p_). Although a moderate correlation can be noted between H_IT_ and a_85_, the inverse correlation between E_IT_ and W_u_ and the total absence of correlation between E_IT_ and either h_max_ or H_IT_ are not surprising.

**Figure 8:**
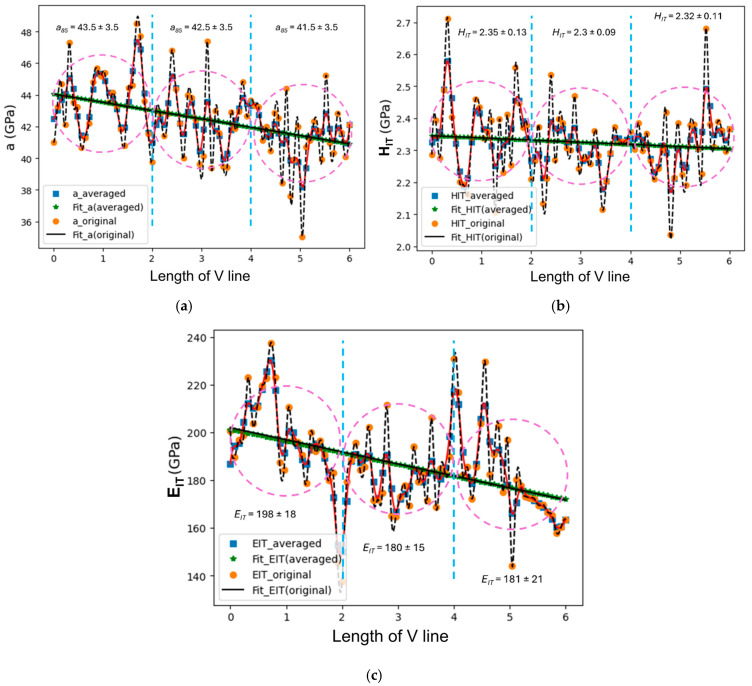
Regressions across the raw data (filled circles) and the average data (Equation (3), filled squares) for a_85_ (**a**), H_IT_ (**b**) and E_IT_ (**c**) along the V line. The black and red dashed fitted lines relate to raw and averaged data points, respectively. The dashed circles delimit the segments partitioned over the V line with the corresponding average values.

The presence of outliers in the raw data posed serious problems for the estimation of the property gradients along the PLs, due to the presence of unphysically large slopes in the respective regression lines. Since the mere systematic cancelation of these outliers could result in misleading results, or a loss of useful material or process-related information, we decided to consider them, in combination with Equation (3), as a suitable averaging method. Figure 8 shows the regressions across the raw data (filled circles) and average data (Equation (3), filled squares) along the V line for a_85_ (a), H_IT_ (b) and E_IT_ (c).

As can be seen, the two regression lines nearly overlap for each property, thereby indicating that the broad trend of each property along the V line is preserved because of the presence of outliers, albeit with less dispersion of the associated standard deviations. For clarity reasons, the V line was divided into three segments of equal length, which implied a division of the plot into three equal regions (see the vertical plot lines), with the leftmost one corresponding to the deposit region near the substrate, the rightmost one to the dome, and the central one to the core of the deposit. An interesting outcome of the above averaging scheme is that a notable trend across the indented layers can now be perceived (blue filled square), especially for LSR. The LSR serves as a guideline to trace the actual trend from among the true indentations (orange filled circles). An interesting periodic change in the indentation properties appears across the bottom region of the deposit, which becomes less marked in the core region and even less appreciable at the dome. Such periodic behavior becomes less noticeable for H_IT_ and E_IT_. LSR exhibits a decreasing trend along the build direction (V line), with a total difference of 5 GPa between the bottom and the top, which corresponds to a 0.8 GPa/mm gradient. If we assume ±3.5 GPa as an average range of the amplitude of the oscillations of the LSR along the V line, the occurrence of the outliers can be seen to be also periodic. Given that about four indents fall into one layer, the first low-outlier is in the middle of the curved Y-scan stripe. The LSR value increases as the V line moves upward (four indentations or ~320 μm) above the first indent, which is located at the bottom of the semi-circle weld pool, where faster cooling is attained [43].

Similar regression lines can be traced for all the PLs (see the Supplementary Material). The regression results are quantitatively summarized in Table 4 for all the PLs but only considering the regression slopes of the raw data. The slopes reveal the severity of the selected L-PBF process conditions, in terms of the gradients of the three major indentation properties across the entire build sample. These slopes are key performance indices that are useful for either the mechanical design of a component and process optimization purposes. Having successfully assessed the reliability of the used averaging method, we used it for the subsequent analysis.

A detailed inspection of Table 4 shows that the gradients of the indentation hardness and LSR are almost negligible along each PL, compared to the indentation modulus. This confirms a close correlation between these two properties and, at least in terms of global trends, the reliability of the calibrated contact area used to estimate the standard indentation hardness. Nevertheless, it is interesting to observe the sign of ∇a_85_, which takes both positive values (along the H1 and D2 lines) and negative values (along the H2, D1, and V lines), with the main negative value being along H2. This highlights that H2 has a specific orientation that should be taken into consideration during the fabrication process, as large differences in the mechanical response may undesirably arise between the two parallel walls of the deposit, particularly at its mid-height. However, after cross linking the results along the H2 and D2 lines (with a pronounced gradient in a_85_), it emerges that E1 is a critical region of the deposit. The most significant gradients of the indentation modulus are observed along the H2 and V lines (negative slope), although the largest gradient is along the H2 line. This additional information indicates E1 as being the softest and most compliant zone in the deposit. Moreover, the H2 direction exhibits the largest standard deviation (see Table 2), which suggests a greater presence of possible heterogeneities, anisotropy or residual stresses, or a combination. The significant value of the indentation modulus gradient along the V line underlines the difference in stiffness between the bottom region (near the substrate) and the top region (near the dome). As the indentation hardness gradients and a_85_ are not equally significant along the V line, other factors may come into play in this case.

**Figure 9:**
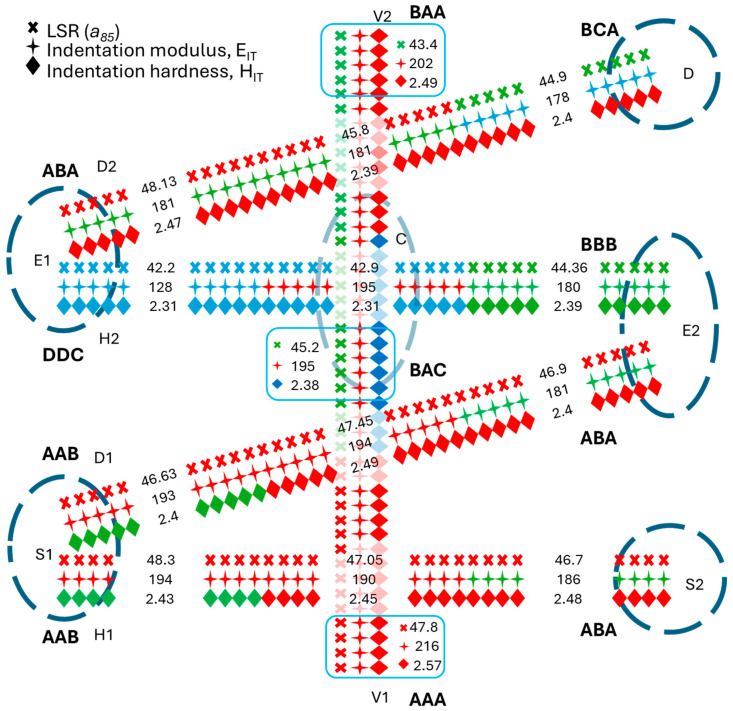
Maximum values per segment of the indentation modulus, indentation hardness and LSR in the six performance zones (PZs, dashed line) extracted from the five-performance lines (PLs). The colors indicate the relative strength (highest level of strength = red, lowest level of strength = blue, moderate level of strength = green).

It is worth noting that when the H_IT_ and LSR gradients were assessed over all the PLs, both properties were closely correlated in sign but seldom in magnitude. Such a poor correlation also arose when Pearson’s correlation method was used. This would seem to suggest that more meaningful correlations may be found over smaller portions of the PLs, such as in PL segments, and this is why the latter were associated with PZs. The maximum values of the three relevant indentation properties were preferred to the statistical averages to improve the correlations between the segment-based indentation properties. Moreover, maximum values better fulfill the lightweight design principle of AM components. Figure 9 becomes relevant for this purpose. Indeed, it sketches the five PLs and the six PZs with the associated maximum values of the indentation properties, H_IT_ (♦), E_IT_ (+) and LSR (×). However, when RS is present in the material, such properties should be intended as lumping properties, which means that, in addition to the microstructure (and its heterogeneities), they also sense the level of residual stress/strain (RS) at the inspected depth. Among these properties, the indentation modules appear to be more sensitive to residual stress than H_IT_ or a_85_. The latter property embodies three stiffness contributions: (a) the stiffness that is responsible for strain hardening across the indentation and which is, in some way, related to H_IT_; (b) the intrinsic stiffness of the material (i.e., Young’s modulus); and (c) the stiffness that is opposed by the internal RS introduced by the manufacturing process (which is conventionally assumed positive for tensile stress and negative for compressive stress). As a result, the RS was sensed in a combined (i.e., indiscernible) manner with the microstructure response during indentation loading and unloading by corrupting the respective indentation curves, which caused an artificial shift with respect to an ideal RS-free indentation curve [44]. Moreover, having considered other factors of influence, such as the micro- and macro-texture, as being less relevant in AM components, we have assumed indentation modulus E_IT_ as the primary indicator of the presence of RS, considering its deviation with respect to the reference Young’s modulus. Any inherent pile-up or sink-in events observed during the indentation of these materials have also been attributed to RS.

In agreement to other works [45], the best hardness and stiffness performances of the deposit were found along its build direction. The three main indentation properties attained the largest value along the vertical V line, except for the CZ, in agreement with the previous analysis on the property gradients. Except for the slightly greater value of E_IT_ (greater than ~190 GPa, the reference Young’s modulus of a 316L alloy) [46], the H_IT_ and LSR values at the CZ were found to be lower than their counterparts at the top and bottom regions of the V line. All the indentation properties at the bottom attained the largest value. These values should be related to the distinctive influence exerted by the substrate at the bottom zone of the V line. Conversely, the lowest values of H_IT_, LSR, and E_IT_ were found along the H2 line, which also presented the largest heterogeneity index (%RSD). Exceptionally low values of all the indentation properties were confirmed for E1(H2). As mentioned earlier on, the local mechanical response of E1 was complemented by the H2 and D2 lines, although E1 (D2) exhibited larger LSR, E_IT_, and H_IT_ values, because different stripes were sensed (i.e., the re-solidified stripes exhibited a relatively high level of hardness and LSR). Because of the different influence of the orientation at the PZs, the mechanical response at each PZ is hereafter specified along with its orientation (if present). Thus, the anomalous E1(H2) led to two consequences, namely, (i) reversion of the property gradients along H2, compared to the H1 line, and (ii) deterioration of the mechanical response at the reference CZ. As the D1 and D2 lines were generally used to trace the indentation properties across multiple layers along with their local microstructure, heterogeneity, and RS, both PLs returned useful information on the selected laser scanning strategy. The almost identical relative standard deviation (%RSD) value along these two lines indicated the presence of similar deposition features, but also different factors at play. Although the D1 line was affected more by the substrate (heat sink and dilution ratio factors), the D2 line reflected the dominant effects from convection cooling on the surface of the dome. However, the D zone exhibited an analogous abnormal drop in E_IT_ to the one observed in E1(H2). The H_IT_ values at the right edge remained nearly constant, whereas E_IT_ slightly decreased toward the top, although it was always below the reference Young’s modulus. This behavior was in contrast with that of LSR, the values of which were fairly good at the right bottom edge and relatively low at its mid-height. The larger values of the indentation properties at the bottom edge (S2) benefitted from the greater influence of the substrate. The H1 and H2 lines traced the indentation properties along the X- and/or Y-scan layers at different heights of the deposit. The earlier stripes along H1 underwent marked carbon dissolution from the melted substrate, while conduction-cooling from the substrate promoted a fine microstructure, due to rapid solidification and re-solidification. Conversely, the stripes along the H2 line were hardly affected by the substrate, while their solidification was controlled entirely by conduction self-quenching, due to the relatively hot surrounding stripes and convection cooling from the edges. A comparison of S1 (H1) and S2 underlined a greater strength and stiffness of the former than the latter. Indeed, the unique isotropic mechanical response of S1 supported the dominant role of the substrate over other external factors. After axially expanding the core region up to D2 and down to D1, we observed a generalized relative softness, which was in contrast with the nearly constant and reasonable LSR, as well as a contrasting indentation modulus between the top (more compliant) and the bottom (stiffer) of the core. Such a complex mechanical response of the core, which resulted from the three indentation properties, would seem to suggest that, in addition to the microstructure, the residual stress–strain also played a role in the mechanical response of the investigated L-PBF 316L build sample. It should be noted that, in the present case, the original self-balanced residual stress–strain state in the L-PBF 316L build had inevitably been altered during the sectioning of the sample, and it had likely undergone further accidental changes during the indentation test. The remaining stress–strain state was generally unbalanced, depending on the selected indentation load. For this reason, we preferred to denote the latter as remnant residual stress (RRS).

## 4. Discussion

### 4.1. The 5PL-nIIT Scheme in the AM Components

The developed 5PL-nIIT scheme was primarily designed to convert arrays of localnanoindentation properties into macro-mechanical responses in specific performance zones that are directly influenced by one or two external system parameters. The expected anisotropy of the AM components suggested a physical-based criterion to generate several indentation arrays (i.e., the performance lines) across the build sample in order to populate the performance zones with the desired macro-mechanical performance. The performance zones were used to establish a direct correlation between the microstructure, the macro-mechanical performance, and the RRS in such key regions which, in turn, could be tuned as a function of the external system parameters. However, the initial self-balanced RS in the original build was able to change to an altered RRS in the sliced sample. This in turn made the determination of the standard indentation properties and their correlation with the external system parameters more challenging or even impossible. In spite of this, the standard indentation hardness and LSR properties are believed to have shared a slight dependence on RS (or RRS), unlike the indentation modulus. The latter was found to be very sensitive to the RS (or RRS) as well as to the micro-texture (which is typically inspected by means of EBSD) and the macro-texture (or fiber texture, which is typically inspected by means of X-ray diffraction).

### 4.2. Microstructure/Anisotropy in L-PBF 316L

Earlier studies on the L-PBF of the 316L alloy clarified the interplay between the thermal gradients and cooling rates with the microstructure morphology [47,48,49,50], and the inherent anisotropy effects of the structure [5,50,51,52]. In this work, no detailed information on the process conditions was available from the producer. Nevertheless, a detailed inspection of IPF in Figure 4a indicates that the longitudinal X-scan layers specifically contributed to the build, under an inherent optimal combination of laser power and scanning rate conditions. The 90° scan rotation was just carried out to force a re-melting/re-solidification of the interlayer regions. Figure 4a shows that as the lower regions of the Y-scan layer tend to recrystallize, the upper region of the X-scan layer induces; (a) breaking of the multilayer columnar growth tendency [53,54], (b) stronger and tougher bonding between the layers, and (c) a smoother layer surface while the residual pores or interstices between the underlying solidified melt pools fill up. The equiaxed grains varied over a few tens of microns. However, we were uncertain whether such Y-scan layers tended to relax the X-scan induced stresses or whether they enhanced them, thereby contributing to the overall greater strength along the build direction. It has been observed that such equiaxed grains tend to increase the strengthening process, according to the Hall–Petch law [55,56], as reported by [57], although faster Y-direction scans in remelting mode promote a lower melt pool temperature, heat flux, thermal gradients, and faster solidification rates. The changes in the indentation properties were in fact appreciable along the D1 and D2 lines, and the LSR values were, on average, clearly large. Thermal gradients may in fact influence the growth rate to a great extent as well as the shape of the cells, which may change into equiaxed or elongated shaped cells [58]. The average size of the cells observed in the present microstructure ranged from 2.5 to 3.5 µm (Figure 5a), whereas the elongated cells may have had the same width, but they had a longer length (~10 µm). The microstructure observed in this study revealed a prevalence of the <101> orientation, intermixed with the <111> orientation, at both the interlayer and intralayer zones. Thus, the employed alternating X- and Y-scans hindered the occurrence of any relevant macro-texture [50] but may have retained some micro-texture, which was detectable by means of EBSD (see Figure 4a) and nIIT. Moreover, the EBSD results showed that large grains often subtended several sub-grains (see the color grading) with expectable local changes in the micro-texture and inherent stiffness. However, we did not expect any appreciable changes in the indentation properties at a macroscale (i.e., build-scale) due to the presence of the macro-texture. Hence, although the indentation modulus may have deviated to some extent (with respect to the reference modulus), especially across large grains, the micro-texture was not able to account for a large portion of the observed large deviations.

### 4.3. RS During IIT

When all the indentation properties over the entire length of the PLs were considered, we were able to estimate the mechanical anisotropy of the build, as provided by the macroscale gradients (*∇_EIT_*) of the three main indentation properties. These gradients accounted for the macro-texture (if present) of the given build along the directions that connected it with its performance zones. The latter are relevant in that they allow the build performance to be tuned by varying a selected set of external system parameters. According to Table 4, the values of the measured gradients were relatively small, but they indicated the presence of the critical directions (e.g., the H2, D1, and V lines), critical zones (e.g., E1(H2), D) and high-performance zones. When the indentation properties over smaller segments extracted from the PLs were considered, we were able to determine the local macro-mechanical response of those performance zones. However, we found that the three extracted indentation properties were inconsistent with each other in certain zones, thereby warranting more in-depth analysis. It is well-known that, in addition to the micro- and macro-texture, macro-residual stresses (MRS) can also affect the global mechanical performance of AM products to a great extent [16] and may even drive the size and morphology of the grains. Prior knowledge of MRS is of paramount importance for engineering design as it can enhance or degrade the performance of an AM product, especially under fatigue service conditions. Indeed, MRS is affected by most of the indicated external system factors, as a function of the internal process parameters (thermal gradients, solidification rate and cooling rates). Thus, differential heating and differential cooling during and after solidification are the main causes of MRS. Microscopic residual stresses, which depend on the precipitated solute segregations being more localized [57], have not been prioritized in this study. Although several methods for measuring RS exist, nIIT benefits from a high versatility in probing the dimensional scale (e.g., penetration depth) and a range of measured properties in the output. Several nIIT methods have been proposed to assess RS in various materials [59]. However, most of these methods suffer from the practical difficulty of finding an equivalent volume of a stress-free material microstructure to the one being tested, especially when microstructural gradients are present across the sample. Indeed, the investigated L-PBF 316L build sample originated from rapid thermal cycling, and severe thermal gradients occurred during solidification, remelting, and re-solidification. The existing MRS, locked into the microstructure, interacted with the localized triaxial stress–strain field exerted by the indenter during the loading and unloading stages. The loading curve tended to shift rightward (greater penetration depths) or leftward (lower penetration depth), depending on whether tensile or compressive RS, respectively, was present, whereas the unloading curve tended to shift in the opposite direction. However, the standard code does not cover the response of such materials in the presence of RS, and its results should be interpreted with great caution. E_IT_ is the most sensitive to RS, as it is estimated from the slope of the unloading indentation curve, whose computation is per se a critical factor, even when dealing with regular materials. With RS, the lack of accuracy in this slope is further magnified due to a change in the unloading curve upon incipient load release. Since this occurrence is quite common in engineering materials, the international code suggests considering a pre-specified portion of the unloading curve to mask abnormal behavior. However, though this expedient can work for slightly stressed materials, it produces misleading results when applied to highly stressed AM products which, among others, may experience opposing RS states in adjacent regions. Moreover, the standard requirement of geometric self-similarity for sharp indenters breaks down in the presence of RS. This may lead to serious implications concerning the evaluation of the contact area, which is usually assumed to be determined from purely elastic contact. The latter requires the material volume underneath the indentation to be fully plasticized and strain hardening to have occurred before the indentation load is released. This is a fundamental requirement to make an indentation test successful in regular elastoplastic materials. The presence of tensile- or compressive-RS reduces when the amount of the necessary plasticity (and subsequent strain hardening) is loaded, and this results in a lower or higher indentation modulus versus the reference Young’s modulus of the material. The ability to probe the elastic properties after strain hardening of the indented region is a major feature of IIT, compared to the uniaxial tensile/compressive test, which may hide subtle material behavior when RS is present. If the expected strain hardening is hindered to some extent during loading, the measured indentation modulus will become sensitive to RS after unloading. For this reason, we considered it reasonable to use such a modulus as an indicator of the RS present in the material. However, it should be recalled that its value may be somewhat inaccurate, especially when a large amount of tensile RS is present. Moreover, relatively intense RS in polycrystalline materials may shield texture effects. In general, the lack of strain hardening in RS-affected materials is accompanied by lower or larger values of H_IT_ and LSR for tensile- and compressive-RS, respectively, than in the absence of RS. The conventional Vickers hardness test is not free from abnormal probing during indentation, as it is inconsistent with H_IT_ and LSR, which may reveal lower levels of hardness (for tensile-RS) and, thus, may erroneously be interpreted as material softening; see, for example, [60,61]. In other circumstances, RS remains undetected or even masked, after a conventional Vicker’s hardness test, as just the mean value of the diagonals of the imprint has to be calculated, regardless of its distortion degree. In this context, IIT is more informative than the conventional hardness test as E_IT_ offers at least a hint on how to detect any possible RS present in the material.

In this work, we have evaluated the influence of RRS on the indentation properties at a considerable depth below the surface. The peak load of 100 mN was selected so as not to be too large to prevent widespread alterations of the RRS, but sufficiently small to cause limited damage while multiple indentations were being made across a layer with a clear sensitivity of E_IT_ to the local RRS [8]. Indeed, locally stable RRS is intrinsically combined with the indentation properties, whereas its relaxation during indentation cycling is typically the cause of their observed deviation.

Despite the material response to an indentation load, via the traditional indentation curves, the material response in terms of stiffness (or load rate) is here proposed to be evaluated by means of the secant stiffness curves. This approach is especially useful when the rate of different mechanisms (e.g., elasticity, plasticity, elasto-plasticity, creep, stress relaxation, or their combinations) need to be identified upon indentation. Even if we are unable to locally measure the RS, the LSR may provide useful information on its stability or susceptibility to change by a sharp indenter. Indeed, a change in LSR on IIT does provide clear evidence of the tendency of RS to relax. As such a tendency is unlikely, we can safely consider the indentation properties of the material as an integral combination of RS and the microstructural response. In this work, we assumed LSR to be an RS-relaxation-free property over a depth (Δh) or load range (ΔF) of the IIT cycle that had to be specified, through the slope (LSR) of the S_h_-h curve. For the investigated L-PBF 316L alloy, such a depth range, which was estimated as ~85% h_max_, means that we neglected any material phenomena that occurred in the initial 15% of the indentation curve in order to determine the indentation properties of the material. We thus found a numerical correlation between LSR and H_IT_. However, it should be recalled that LSR, unlike H_IT_ and E_IT_, does not depend on the area function (calibration), which, per se, introduces additional sources of inaccuracy into the standard indentation properties when RS is involved. Thus, LSR can be viewed as a lumping ISE-free property that may combine mechanical effects from the microstructure, micro-texture, and from the RS.

The investigated L-PBF 316L sample was cut from an original build using electro discharge machine. The longitudinal RS, induced by the fabrication process along the X-scan direction, was considered much larger than that in the Y-direction, due to the longer scan vector, but it was considered negligible after RS relaxation, due to sample slicing [16]. Since no cracks were observed during slicing, the RRS in the sample was hypothesized to have returned to a self-equilibrated state at a lower peak level. Nanoindentations were designed over the Y-Z plane to assess the microstructure and the inherent RRS effects along the X direction.

The mean values of the indentation hardness (or its equivalent Vickers hardness) listed in Table 2 are consistent with those found in the literature [13,61,62], although they account for a partial relaxation of the initial RS. Considering the large spectrum of external parameters of influence investigated in the literature, it is surprising that such a consistency in properties can be observed. There are two possible reasons for such a consistency: (a) the tested material may only have been affected marginally by RS (e.g., very shallow indentation peak loads), (b) the hardness definition may not have been so sensitive to the anomalies of the material, such as RS or texture (this does not apply to Knoop hardness), (c) the preparation of the sample may have completely erased the anomalies. Indeed, indentation hardness may not be useful to fully discriminate changes in AM components. However, data on indentation moduli is quite rare in the literature.

### 4.4. PZ with Critical Mechanical Performances: E1(H2) and D

The maximum value of the observed abnormal indentation modulus (128 GPa in Figure 9) was determined in the E1(H2) zone by partitioning the H2 line into three segments, each with equal number of indents, and considering the maximum value of the pertinent segment. This segment was approximately 1/3 of the deposit width. This underlines that dealing with an anomaly is not a local issue, and it instead concerns a considerable portion of the sample and affects all the indentation properties. As the E1(H2) zone was located in the transition region, where the planar edge bent into a curved dome, its critical mechanical performance was ascribed to an accidental hot spot that originated during the deposition of the Y-scan layers (the shorter deposition scan vector, equal to the deposit width) due to an insufficient heat dissipation time. The vicinity of the E1(H2) zone to the leading edge of the deposit accentuated such an adverse local condition even more. Indeed, the deposition area at the curved dome progressively reduced as the build height increased until it reached its highest point. This rationale infers that (a) the E1(D2) zone, i.e., the same E1 zone along the D2 line, with the D2 line detecting multiple X-scan and Y-scan layers, is nearly free from such an anomaly. Exception can be made for the slightly lower local stiffness (181 GPa), which is a common feature of the entire outline of the sample; (b) the topmost D zone (near the curved dome surface) shares a similar anomaly with E1(H2), although it is less severe. Overheating phenomena are commonly encountered in large hexagon- or island-pattern scans where heating frequently results in excessive peak temperatures, localized cell growth and severe tensile RS, due to the restraints being opposed by the surrounding colder material [16,63]. Thus, the larger the local tensile-RS is, the larger the expected deviation of the indentation modulus from the reference modulus (190 GPa). In our case, the RRS could not be released on fabrication because of the stiff substrate and the absence of micro–macro porosity [63]. Accordingly, the relatively low value of the indentation modulus found in E1(H2) indicates that a quite large tensile-RRS was also responsible for the dramatic drop in the LSR and H_IT_ values. The latter resulted from the poor plasticity and strain hardening that occurred during indentation loading. The abnormal behavior of the H2 line also emerges from the large %RSD in the E_IT_ (see Table 2) which denotes severe levels of heterogeneity along that direction. Such variations show that the nano-IIT was highly sensitive to the heterogeneities in the tested samples [64,65]. In short, the E1(H2) and D zones were affected by a concurrent drop in all the indentation properties, due to the presence of detrimental tensile-RRS states.

### 4.5. PZ with an Outstanding Mechanical Performance: Deposit Root

The stress condition at the bottom of the V line (adjacent to the substrate) was reversed to the right with respect to the E1 zone. A pure compressive-RRS originated in this area, as shown by the larger local indentation modulus (216 GPa) than the reference modulus (190 GPa), which was supported by the large values of the other indentation properties (LSR = 47.8 and H_IT_ = 2.57 GPa). According to [66], the cool-down phase of the top molten layers tends to thermally contract, and this deformation is restrained by the stiff-cold substrate, which imparts a tensile stress state to the top layers and a compression stress state to the substrate. This mechanism is replicated in the same way for each upper layer, with respect to the underlying colder layer. In the present case, the substrate was quite stiff (high Young’s modulus and greater thickness) and strong, thereby binding the peak of the induced RS [16].

### 4.6. PZ with an Indefinite Mechanical Response: CZ

The E_IT_ values in the CZ appear to be inconsistent with the H_IT_ and LSR values. This zone is located at the crossing point between the two main deposit lines, that is, the H2 and V lines. The CZ can capture the external changes imparted to the build through most of the external system parameters. Apart from the dome-tip region, E_IT_ also varied along the V line, where good values were attained in the CZ and in its sub-core (195 GPa), while fair values were observed at the H1 line (190 GPa), although the largest value was observed at the base of the deposit (216 GPa). These values indicate a favorable stiffness performance along the deposition direction, we were not able to infer whether that status strictly reflected the build in the as-fabricated condition, as the original RS had been at least partially relaxed during finishing, sample preparation and in the indentation tests. Consistent with these findings, many reports have stated that the build direction determines the main performances after L-PBF parts have been tested [57,67]. However, the full set of nanoindentation results shown here indicate that H_IT_ did not follow the E_IT_ trend along the V line, and the LSR trends, which were often consistent with the H_IT_ ones, deviated from that of E_IT_ at the deposit core (CZ) and its sub-core. However, the three indentation properties were consistent with each other at the deposit base, where the substrate imparted outstanding strength and stiffness to the deposit. However, such a complete matching between the indentation properties no longer held at the CZ(H2) zone, where the good values of E_IT_ conflicted with the low values of LSR and H_IT_. Two competing mechanisms are believed to have occurred at the mid-height of the deposit (i.e., the critical H2 line), and these may help explain the local anomaly that was observed. The E_IT_ values that existed along the build direction may be considered as indicators of a more severe three-dimensional self-equilibrated RS of the as-fabricated deposit, which had a much greater initial compression-stress component in the Z-direction that then partially relaxed after sample slicing, as can be seen from the resulting RRS along the H2 and V lines. The interaction between both RRS states resulted in a deteriorated indentation response of the CZ. This rationale can be substantiated by considering the values of LSR, which were relatively small in the CZ (42.9 GPa), but were not so small in the sub-core region (45.2 GPa) and upper core (45.8 GPa). This difference indicates the existence of a contrasting mildly compressive RRS state along the build direction, while there was a severe tensile-RRS component in the transversal direction. The latter can be attested by the low values of 2.31, 2.38 and 2.39 GPa, respectively, which were observed in the regions covered by the H2 line. The severe tensile-RRS, which was located along the H2 line, was further confirmed by the low values of H_IT_, but not by the values of LSR, because the latter was the lumping indicator of the hardness and stiffness. Nevertheless, it is currently not possible to obtain real proof of the correlation between the two conflicting RRS states with the current high values of E_IT_ and low values of H_IT_, and this indicates the need for further in-depth studies in this direction. Moreover, it is worth pointing out that the standard indentation properties and LSR combine with the local RRS during an indentation test. Although the former primarily depend on the single value of h_max_ (and to a lesser extent on the contact area, which may deviate from the assumed pure elastic one), the latter senses the evolution of RRS, together with the elastoplastic phenomena, during the entire loading stage up to h_max_. As LSR is represented by the slope of the secant stiffness curve, it returns the hardness index, via the h_max_ value, and, more importantly, the lump stiffness of the material. Here, lump denotes the two stiffness contributions that are encountered during indentation loading, that is, the intrinsic stiffness of the material (Young’s modulus) and that associated with material strain hardening. However, discerning these two contributions from the indentation properties can be cumbersome to some extent in materials that contain both compressive and tensile RS. Moreover, the possible influence of individual crystal orientations on the measured indentation modulus imparted by the relative presence of <101> orientated grains across the core of the deposit cannot be excluded. Indeed, although the observed grains were quite large in the L-PBF 316L alloy, the PZs may have encompassed several grains, so that these grains, mediated by the surrounding grains, may have contributed to their mechanical response.

### 4.7. PZ with an Outstanding Mechanical Response: S1

The substrate (AISI 1020) at the base of the deposit contributed by enhancing the strength and stiffness of the adjacent deposited layers. The contraction of the adjacent layers upon solidification was restrained at the interface between the substrate and the deposit. The expected warping was hindered by the stiff substrate, which generally sets RRS at the interface between the substrate interface and the deposit, and may also operate as a driving force of changes in the grain morphology [63]. The substrate affected the deposit layer through two mechanisms: (a) carbon enrichment (i.e., chemical dilution) in the liquid phase, and (b) severe conduction cooling (the heat sink effect). The carbon that diluted into the first layers directly impacted the local indentation properties via solid solution strengthening, but also by increasing the heterogeneity level H1 (see the %RSD of E_IT_ in Table 1). These characteristics highlight the mechanical response of the deposit base, especially at the V centerline, where we observed the largest indentation properties. The mechanical response of the two opposing PZs, that is, S1 and S2, could be attributed to different local temperatures, as one region was part of the leading edge and the other belonged to the trailing edge. Since the temperature along a stripe increases as the scan vector length and number of layers increase [68], the two regions must have experienced quite different local temperatures during fabrication. Some authors have computed RRS in an SLM IN718 alloy [43,69]. These authors found a larger heat conduction dissipation to the substrate at its centerline, which culminated in the following RS state: (a) larger σ_x_ of the tensile type at the center and highly compressive toward the edges; and (b) σ_y_ overall smaller than σ_x,_ but with almost the same trend; σ_z_ entirely compressive along the interface, with a possible tendency toward a tensile state close to the edges. The mechanical responses found in the S1 and S2 regions appeared to be consistent with the above research, that is, the largest compression state of σ_z_-RRS was observed at the centerline (see the largest E_IT_ = 216 GPa), while a less compression state was seen in the left corner S1(H1) (see E_IT_ = 195 GPa), and a less tensile state (σ_z_) was observed in the S2(H2) corner (see E_IT_ = 186 GPa). As the local temperature at the leading edge of S1(H1) was expected to be lower than at the trailing edge of S2(H1), its microstructure was finer, and its mechanical response was greater in the former corner than at the latter one. Specifically, this was true for E_IT_ and LSR, but, surprisingly, did not hold for H_IT,_ which was lower in S1(H1). Conversely, although LSR followed the E_IT_ trend, by proportionally decreasing its value from S1 to S2, H_IT_ showed an opposite tendency, which means this property may fail to give reliable results when severe RS alters the nature of the contact at the interface between the material and the indenter, which is considered to be purely elastic [24].

### 4.8. Gradient of Indentation Properties

The Pearson correlation coefficient (Table 3) determined a weak correlation between H_IT_ and E_IT_. This is a somewhat paradoxical result in the absence of RS, as the L-PBF 316L alloy can be expected to become hard after strain hardening indentation, and it is an intrinsically stiff material. Table 1 shows that E_IT_ was affected mostly by heterogeneities in nearly all directions (PLs), especially along the critical (most compliant) H2 line and the stiffest V line. Such heterogeneities may include all the features that make the indentation properties, and especially the indentation modulus, particularly sensitive to their presence (e.g., RS, macro-texture, and fabrication defects which, however, were minimal in this case). If we neglect the peripheral deposit regions, where <001> orientated grains may have survived after multiple laser passes, especially for the 90° rotation scan strategy that was adopted in our case, neither exceptionally low nor exceptionally high values of the indentation modulus can be expected across extensive macro-regions, such as the PZs. This clearly suggests that macro-RRS, rather than a micro- or macro-texture, is a dominant factor which influences the mechanical response of a material in PZs. The detected mechanical responses along the V and H1 lines, in terms of all the indentation properties, clearly confirm the presence of RS, as induced by the interplay between the thermal gradients and the cooling rate during the fabrication process. The regression fit of the three indentation properties along the five PLs shown in Table 4 indicates the slopes or ∇E_IT_ gradients. It can be seen from a comparison of these slopes that ∇E_IT_ undergoes the most changes in all directions, thereby underlining its high sensitivity to RRS. The low negative value of ∇E_IT_ along the V line indicates the important presence of a compression-RS state at the bottom of the deposit. Conversely, the lowest negative value of ∇E_IT_ along the H2 line confirms the presence of a marked tensile-RRS in the faulty E1(H2) zone. The increase in ∇E_IT_ along H1 and D2 (i.e., between the opposing parallel edges), implies a comparatively stiffer and stronger left edge (except for the E1(H2) zone) with respect to the right one, thereby underlining an uneven thermomechanical condition, as obtained from the selected scanning strategy.

### 4.9. The Role of LSR (a_85_) as a Mechanical Performance

**Figure 10:**
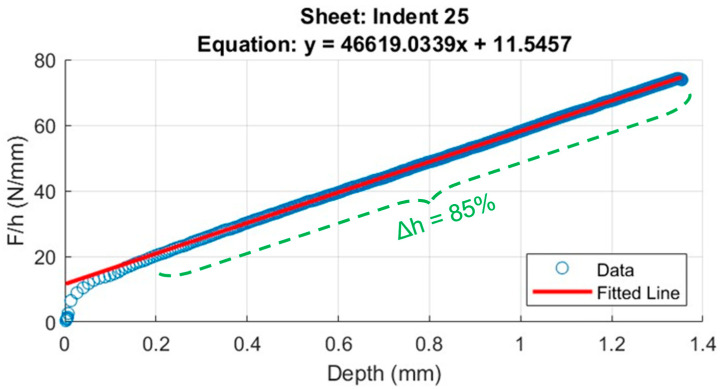
A typical Sh vs. h plot for the given L-PBF 316L deposit. The linear steady state is nearly 85% of the maximum indentation depth.

LSR has been introduced in this work as a supplemented ISE-free material property to help discriminate the eventual presence and any signs of RRS. We have achieved this by cross-linking it with the standard indentation properties, that is, H_IT_ and E_IT_. LSR enables the hardness and the stiffness of a material to be sensed at the same time, without requiring any calibration or prior assumptions. The 85% validity range estimated for the L-PBF 316L alloy indicated its invariancy over quite an ample depth range of h_max_. Figure 10 shows a typical S_h_ vs. h curve derived from the L-PBF 316L deposit. The local secant stiffness varied linearly over a quite extensive depth (or load) range towards the maximum whereas the lowest indentation range suffered dominant non-linear surface anomalies including ISE. The %RSD values of LSR shown in Table 2 point out that this property is affected less by heterogeneities than H_IT_ and E_IT_, which would seem to indicate that if it were affected by RRS, they would be connected to this property, which could hence be used as a reliable mechanical response of the material. The nearly constant values of the b_85_ factor (the *y*-axis intercept of the secant stiffness straight line) and its small %RSD further confirms the very good quality of the regression of this property for the investigated alloy. It should be recalled that LSR includes three on-loading stiffness contributions: (a) intrinsic stiffness of the material (i.e., Young’s modulus), (b) the stiffness associated with strain hardening of the indented region, and (c) the stiffness associated with RS (which is assumed elastic in nature). According to Table 4, the gradients of this property (∇a_85_) reveal negligible changes along the five PLs. However, it highlights a sign change along the H2, D1 and V lines, which have been considered as prominent directions that are affected by RRS. Moreover, ∇H_IT_ exhibits even smaller variations than ∇a_85_, but shares the same sign with it along the H2, D1, and V lines. This suggests that both properties, despite their common dependence on h_max_, incorporate the contribution of RRS, but account for it in a different way. Indeed, the calibration of the area function of the indenter requested by H_IT_, in compliance with the standard code, introduces a new source of deviation from LSR. The maximum values of LSR shown in Figure 8 vary from the lowest value of 42.2 for E1(H2) to the highest of 48.3 GPa for the S1(H1) corner. In contrast to E_IT,_ the LSR values along the H1 line (near the deposit–substrate interface) were very high, which proves the dominant role of the substrate in the L-PBF of the 316L alloy. On average, LSR exhibited a decreasing trend, thereby indicating a progressive reduction in conduction cooling from the substrate as the deposit height increased up to the H2 line in favor of a less severe cooling condition, due to radiation/convection (over an increased surface area) and fewer re-heating cycles. The inherently lower values of both the cooling rate and of the gradient-to-solidification rate ratio at the dome may explain the lower and less widespread values of the LSR. The latter was also found to vary periodically across the deposited layers, especially at the lower part of the deposit, hence discerning the greater strength/stiffness of the unaltered regions inside the semi-circle (melt pool) layers from the regions inside the curved melt pool layers. The E1(D2) zone above the H2 line presents a minor anomaly, which is characterized by reasonably good values of H_IT_ (2.39–2.47 GPa) and by slightly tensile-RRS (E_IT_ = 181 GPa) compared to an almost-outstanding LSR (48.13 GPa). Moreover, the LSR along the D2 line is relatively high, but smaller than the LSR along the H1 line. According to Figure 2, the D2 line crosses the dome region diagonally (at nearly 45°). Apart from the slight tensile-RRS expected at the dome surface, the high values of the E1(D2) should be associated with a large variability of the indentation properties along D2. According to Table 4, the ∇a_85_ gradient is larger along D2 (0.09), with increasing values toward the left leading edge. Moreover, with reference to Table 2, LSR attains large average values along the H1, D1, and D2 lines. LSR suggests that, apart from H1, which is affected by the substrate phenomena, the other two lines (D1 and D2) sense the deposit across the various overlapped stripes. This may result in microstructural features with the highest indentation properties across the deposit. The two diagonals, D1 and D2, exhibit the highest LSR values, which are much larger than those along the V line. This means that D1 and D2 have outstanding strength-stiff (lamping) properties, as defined by the LSR. Conversely, the V line exhibits outstanding compressive-RSS buildup properties, as expressed in terms of E_IT_. Thus, the problem of RRS (or RS), and particularly their phenomenological separation from the true mechanical properties, is very difficult to solve in AM products. Indeed, even the finite element models that are frequently invoked to support AM experiments, and/or complex material testing, cannot alleviate this problem, as the input material properties used in such models are naturally affected by the unknown RRS, thereby preventing their appropriate experimental validation. In this work, we have instead seen that ISE-free LSR measured on loading may exhibit RRS-affected strength/stiffness of a material, just like tensile properties do [70]. Thus, if the plastic relaxation of RRS on loading is negligibly small (e.g., <15% here), the measured LSR can be proposed as a new index for the mechanical design of AM components. Moreover, if the measured contact area is reliable, it can be used to convert the LSR into an equivalent H_IT85_.

**Figure 11:**
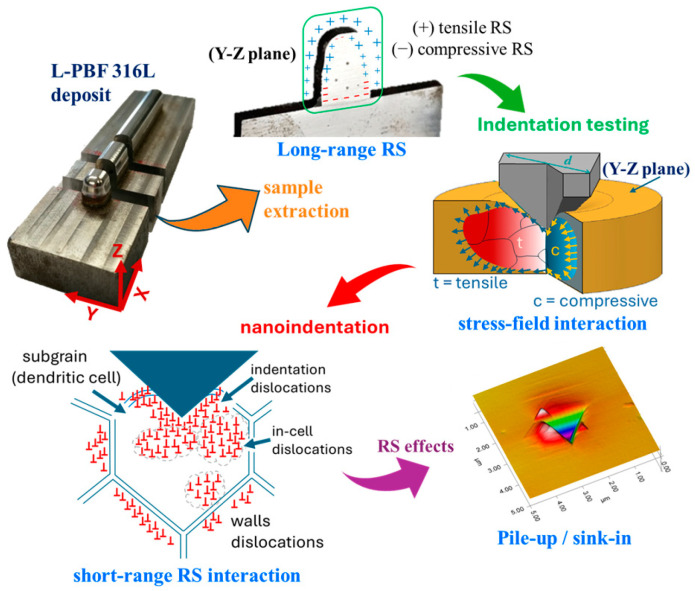
Schematics overview of the methodology followed in this paper. The interaction between locked short−range and long−range residual stresses with the indentation stress−strain field leading to the indentation performance in the L-PBF 316L deposit is depicted.

Figure 11 summarizes in a pictorial fashion the presented methodology. It shows the interaction between the long−range and short−range RS locked in the L-PBF 316L deposit with the stress field induced by the indentation. Upon cutting the initial deposit, the built−in longitudinal residual stresses undergo partial relaxation while maintaining the in-plane residual stress field induced by the substrate. Residual stresses can be of either tensile (+) or compressive (−) in nature. Long−range residual stresses were accounted for by the indentation properties gradients measured along the PLs. The local indentation properties intrinsically included the locked−in residual stress as the latter could neither be discerned nor directly measured. 

### 4.10. Ranking of the Mechanical Performance of an L-PBF 316L Deposit

The 5PL-nIIT scheme allowed us to define a few PZ, to which we assigned the mechanical performance as a function of the three indentation properties. In practical situations, as in the case of benchmarking or process optimization operations, rather than providing the precise values of such performances, a qualitative measure of them can be sufficient. The numerical values (mean values or maximum values) of each indentation property group four individual performance indices (PI) to express the level of strength & stiffness under safe conditions in any PZ. The performance index may take on the following levels, A = outstanding & safe, B = good & safe, C = fair, D = critical & failure. A PZ is denoted as safe-performing when all three of the indentation properties (X-X-X) are specified as safe, otherwise it is denoted as uncertain or unsafe. The safe-performing status is composed of (at least) one pair of high indices of the A-A or A-B type for two indentation properties, without any low index (C or D) for the third property. An uncertain status can be turned into a safe-performing status by fine-tuning some of the parameters of the system or by refining the part geometry. On the other hand, an unsafe status can only be turned into a safe-performing status after heavy re-designing of the part and/or after a complete re-setting up of the fabrication process. Accordingly, the mechanical performances of the various PZs, as shown in Figure 9 for the case of the analyzed L-PBF 316L alloy, may be ranked according to four ranges, which are defined for each indentation property as listed in Table 5. These ranges are defined assuming a tolerance of 0.5 and 0.1 GPa for LSR/E_IT_ and H_IT_, respectively, for the given L-PBF 316L alloy.

Thus, the reference PI (XXX) of any critical or outstanding region can readily be identified by composing the individual PIs for the three indentation properties, so that certain remedies can promptly be undertaken to appropriately balance them across the given AM deposit. The base- and the top-centerline regions, together with the S1, S2, and E2 zones, ranked good or even higher performances and safe levels for the investigated build sample. Conversely, the CZ, with its ambiguous performance status, needs appropriate improvements to mitigate the building up of excessive local tensile RRS, which has been associated with the fabrication of the dome, along the transversal direction of the deposit. It is believed that the tuning of the inherent deposition parameters could solve the problem, not only in this zone but also in the most critical E1 zone, which deserves more attention, and in the D zone. However, since we are unaware of how the original RS is altered as a result of sample slicing, efforts need to be made to properly modify the component design, its fabrication, and the sample holder to permit nanoindentation tests of the original 3D component to be made. The PIs associated with each PZ of the deposit are presented in Table 6.

Moreover, the Y-scan deposition along the width direction of the sample was found to contribute to the temperature differences between the leading and the trailing edge; an appropriate tuning of the deposition parameters may improve the property gradients across the build.

## 5. Conclusions

The indentation properties measured in the L-PBF 316L deposit are intrinsically dependent on residual stresses which are generally pointwise variable. The measured indentation properties should be regarded as indentation performances and care should be taken when comparing them across the tested surface. Nevertheless, the concept of indentation properties was maintained through the paper to distinguish them from the true local indentation performances measured in the performance zone.

The designed PL-nIIT scheme offers an efficient testing strategy that can be used to assess the mechanical performances in terms of LSR, E_IT_, and H_IT_ properties. The special symmetry of the investigated L-PBF 316L deposit and the employed 90° alternating X-scan and Y-scan strategy suggested the definition of five performance lines and six performance zones. The local indentation performances in the performance zones were determined as the maximum values over a subset of indentation properties measured along the performance lines and accounted for combined effects of microstructure, anisotropy, and both short-range and long-range residual stresses. The local performances of the PZs present along the boundary of the deposit revealed the effect of the system parameters associated with each of them. The gradients of the three indentation properties revealed the degree of asymmetry in hardness and stiffness distributions developed between the two lateral edges of the deposit across the layers.

The presence (intensity and nature) of residual stress in each PZ was mainly associated to the deviation between the local indentation modulus and the reference Young’s modulus of the alloy (which was assumed 190 GPa). The identification of pure tensile residual stress in the indentation performances was obvious when LSR, E_IT_, and H_IT_ values were all relatively small (and conversely for pure compressive residual stress). Consistency among the indentation properties was first proved by means of the Pearson correlation coefficient, which indicated a poor correlation between H_IT_ and E_IT_, because of the presence of long-range residual stress. Incidentally, the observed relative standard deviations (%RSD) was relatively large for E_IT_ (6.1–18.6) along the different PLs because of the presence of residual stress. A new indentation property, the loading stiffness rate (LSR or a_85_), was purposely introduced to better discern the presence of residual stress. It is a lumping ISE-free indentation property albeit it embodies hardness and stiffness contribution along with residual stress and grain anisotropy effects. The significance of LSR was determined within the 85% of h_max_ whereas the remainder 15% was associated with typical superficial anomalies, residual stress and ISE of the investigated L-PBF 316L deposit.

The greater hardness/stiffness detected across the L-PBF 316L deposit was due to mild compression residual stress along the axial (build) direction. This resulted from the positive deviation between the indentation modulus and Young’s modulus at the deposit root and at the subsurface top dome (216 and 202 versus 190 GPa, respectively). Compression at the root was promoted by severe conduction cooling and carbon dilution from the substrate whereas that at the top dome was due to fast convection cooling from the edges. Accordingly, the LSR and H_IT_ decreased as the build height increased from 47.8 to 43.4 and from 2.57 to 2.49 GPa, respectively. Two unsafe zones (E1 and D) in the deposit suffered from severe intense tensile residual stress as revealed by the lowest values of all indentation properties (42.2, 128, and 2.31 GPa) and (44.9, 178, and 2.4 GPa), respectively. Both zones were likely to have been affected by hot spots during fabrication. Interestingly, the entire horizontal H2 line, at the deposit mid-height, exhibited an obvious tensile stress state except for the core zone. It is not clear whether such an abnormal state originated from the fabrication of the top dome, due to the sudden shrinking of the deposit layer, or from the sudden change in the deposit cooling regime (i.e., from conduction cooling by the substrate to convection cooling by the edges). The core zone of the deposit, where all the system parameters played a role albeit in a mitigated manner, was assigned as reference region. It revealed an ambiguous mechanical response among the three indentation performances (42.9, 195, and 2.31 GPa). We ascribed this conflicting hardness/stiffness state to the interaction between two opposing stress states across the deposit, specifically between the compressive-σz component acting along the axial direction and the aforesaid tensile-σy component along the H2 line. A qualitative ranking criterion was finally proposed to index the levels of mechanical performance at PZs. This may be useful for qualitative benchmarking or to readily identify the zone requiring special attention for engineering design or reprocessing. The ISE-free LSR is believed to be particularly useful in multiple-load indentation tests of AM parts.

## Figures and Tables

**Table 1:** Weight percentage of the elements found in the L-PBF 316L deposit.

Elements	Si	Mo	Cr	Mn	C	Ni	Fe
% wt	0.91	2.34	18.05	1.57	0.03	12.3	Balance

**Table 2:** Mean values of the PLs and RSDs of the indentation hardness, indentation modulus, maximum penetration depth, loading stiffness rate (a_85_), surface anomaly factor (b_85_), and the power law parameters of the loading curves (K_L_, n_L_).

	Near the Substrate		Near the Dome		Depositing Height
Direction of the Lines	H1	D1	H2	D2	V
H_IT_ (GPa)	2.32 ± 0.12	2.30 ± 0.12	2.26 ± 0.11	2.30 ± 0.11	2.32 ± 0.11
(%RSD)	(5.1)	(5.2)	(4.9)	(4.8)	(4.7)
E_IT_ (GPa)	180.3 ± 11.0	178.8 ± 11.8	152.2 ± 28.3	171.1 ± 10.0	186.8 ± 20.1
(%RSD)	(6.1)	(6.6)	(18.6)	(5.8)	(10.7)
h_max_ (nm)	1372.4 ± 35.9	1377.9 ± 32.9	1398.6 ± 35.1	1381.4 ± 32.4	1367.9 ± 30.9
%RSD	(2.6)	(2.4)	(2.5)	(2.3)	(2.2)
K_L_	526.6 ± 172.6	580.6 ± 147.9	510.9 ± 262.0	492.7 ± 110.5	639.8 ± 210.9
(%RSD)	(32.7)	(25.4)	(51.2)	(22.4)	(32.9)
n_L_	1.7 ± 0.04	1.67 ± 0.036	1.7 ± 0.04	1.7 ± 0.03	1.66 ± 0.04
(%RSD)	(2.6)	(2.2)	(2.4)	(1.7)	(2.7)
a_85_ (GPa)	44.5 ± 2.9	44.36 ± 2.2	42.0 ± 2.2	44.3 ± 2.2	42.5 ± 2.3
b_85_ (N/m)	14.8 ± 2.4	14.33 ± 2.4	15.6 ± 2.2	14.4 ± 1.7	17.2 ± 2.5

**Table 3:** Pearson’s coefficient correlation values for the one-to-one relationship between the most relevant PL indentation indices. Values close to either +1 or −1 indicated a close direct or indirect correlation respectively, between the two quantities.

PLs		W_u_	W_p_	W_t_	h_max_	a_85_
H1	H_IT_	0.041	−0.87	−0.87	−0.99	0.77
H2		−0.1	−0.75	−0.81	−0.95	0.68
D1		−0.07	−0.83	−0.84	−0.99	0.68
D2		−0.21	−0.86	−0.87	−0.99	0.86
V		0.26	−0.81	−0.77	−0.97	0.6
H1	E_IT_	−0.73	−0.45	−0.54	−0.58	0.42
H2		−0.9	0.08	−0.42	−0.44	0.46
D1		−0.83	−0.31	−0.45	−0.5	0.4
D2		−0.81	−0.42	−0.5	−0.61	0.50
V		−0.83	−0.05	−0.23	−0.14	0.35
H1	h_max_	0.04	0.87	0.87	1	−0.77
H2		0.35	0.66	0.86	1	−0.75
D1		0.15	0.83	0.84	1	−0.68
D2		0.29	0.85	0.88	1	−0.85
V		−0.12	0.84	0.83	1	−0.65

**Table 4:** Slope of the regressions (i.e., gradients ∇) of the raw data properties along the PLs.

	H1	H2	D1	D2	V
∇a_85_	0.08	−0.06	−0.02	0.09	−0.04
∇H_IT_	3.1 × 10^−3^	−1 × 10^−3^	−5 × 10^−4^	2.6 × 10^−3^	−5 × 10^−4^
∇E_IT_	0.34	−1.62	0.31	0.14	−0.4

**Table 5:** Ranges of the indentation properties used to define the performance indices of the PZs identified in the L-PBF 316L deposit.

	A	B	C	D
a_85_ (GPa)	≥46.5	[44.5–46.5]	[44–43]	<43
E_IT_ (GPa)	>191	[180.5–190.5]	[175–180]	<175
H_IT_ (GPa)	>2.40	[2.35–2.39]	[2.2–2.34]	<2.2

**Table 6:** Performance indices for the regions of the L-PBF 316L deposit. The meaning of these indices is given in the text and the corresponding values are reported in Table 5.

Base-Centerline	S1(H1, D1)	E1(D2) S2(H1) E2(D1)	Top-Centerline	CZ(H2, V)	E2(H2)	D(D2)	E1(H2)
AAA	AAB	ABA	BAA	BAC	BBB	BCA	DDC

## Data Availability

The data presented in this study are available in the article.

## Supplementary Materials

The following supporting information can be downloaded at: https://www.mdpi.com/article/10.3390/ma18071462/s1, Figures S1–S4: Regressions across the raw data (filled circles) and the average data (filled squares) for indentation properties along the D1, D2, H1 and H2 performance line, respectively.

## References

[1] Hamidi M., Leuven N.K., Nasab M.H., Loge R., Casati R. A Study on the Surface Features of the SLM Processed Parts and Their Journey to Become Bulk Defects. Proceedings of the Euro PM 2019 Congress & Exhibition, European Powder Metallurgy Association (EPMA).

[2] Ramirez-Cedillo E., Uddin M.J., Sandoval-Robles J.A., Mirshams R.A., Ruiz-Huerta L., Rodriguez C.A., Siller H.R. (2020). Process planning of L-PBF of AISI 316L for improving surface quality and relating part integrity with microstructural characteristics. Surf. Coat. Technol..

[3] Wang X., Muñiz-Lerma J.A., Sanchez-Mata O., Atabay S.E., Shandiz M.A., Brochu M. (2020). Single-crystalline-like stainless steel 316L with different geometries fabricated by laser powder bed fusion. Prog. Addit. Manuf..

[4] Im Y.D., Kim K.H., Jung K.H., Lee Y.K., Song K.H. (2019). Anisotropic Mechanical Behavior of Additive Manufactured AISI 316L Steel. Metall. Mater. Trans. A Phys. Metall. Mater. Sci..

[5] Charmi A., Falkenberg R., Ávila L., Mohr G., Sommer K., Ulbricht A., Sprengel M., Neumann R.S., Skrotzki B., Evans A. (2021). Mechanical anisotropy of additively manufactured stainless steel 316L: An experimental and numerical study. Mater. Sci. Eng. A.

[6] Ni X., Kong D., Wen Y., Zhang L., Wu W., He B., Lu L., Zhu D. (2019). Anisotropy in mechanical properties and corrosion resistance of 316L stainless steel fabricated by selective laser melting. Int. J. Miner. Metall. Mater..

[7] Kong D., Ni X., Dong C., Zhang L., Man C., Cheng X., Li X. (2019). Anisotropy in the microstructure and mechanical property for the bulk and porous 316L stainless steel fabricated via selective laser melting. Mater. Lett..

[8] Maizza G., Hafeez F., Varone A., Montanari R. (2024). Nanoscale and Tensile-Like Properties by an Instrumented Indentation Test on PBF-LB SS 316L Steel. Materials.

[9] Sun Z., Tan X., Tor S.B., Chua C.K. (2018). Simultaneously enhanced strength and ductility for 3D-printed stainless steel 316L by selective laser melting. NPG Asia Mater..

[10] Suwas S., Ray R.K. (2014). Texture and Properties.

[11] Sun Z., Tsai S.P., Konijnenberg P., Wang J.Y., Zaefferer S. (2024). A large-volume 3D EBSD study on additively manufactured 316L stainless steel. Scr. Mater..

[12] Uddin M.J., Ramirez-Cedillo E., Mirshams R.A., Siller H.R. (2021). Nanoindentation and electron backscatter diffraction mapping in laser powder bed fusion of stainless steel 316L. Mater. Charact..

[13] Kurdi A., Tabbakh T., Basak A.K., Au A.K.B. (2023). Microstructural and Nanoindentation Investigation on the Laser Powder Bed Fusion Stainless Steel 316L. Materials.

[14] Andreau O., Koutiri I., Peyre P., Penot J.-D., Saintier N., Pessard E., De Terris T., Dupuy C., Baudin T. (2019). Texture control of 316L parts by modulation of the melt pool morphology in selective laser melting. J. Mater. Process. Technol..

[15] Terner M., Lee J., Marchese G., Biamino S., Hong H.U. (2020). Electron Backscattered Diffraction to Estimate Residual Stress Levels of a Superalloy Produced by Laser Powder Bed Fusion and Subsequent Heat Treatments. Materials.

[16] Bartlett J.L., Li X. (2019). An overview of residual stresses in metal powder bed fusion. Addit. Manuf..

[17] Voyiadjis G.Z., Almasri A.H. (2009). Variable Material Length Scale Associated with Nanoindentation Experiments. J. Eng. Mech..

[18] Birnbaum A.J., Ryou H., Steuben J.C., Iliopoulos A.P., Wahl K.J., Michopoulos J.G. (2020). Nested size effects in the nanoindentation response of additively manufactured 316L stainless steel. Mater. Lett..

[19] Pöhl F., Hardes C., Scholz F., Frenzel J. (2020). Orientation-Dependent Deformation Behavior of 316L Steel Manufactured by Laser Metal Deposition and Casting under Local Scratch and Indentation Load. Materials.

[20] Čech J., Haušild P., Kovářík O., Materna A. (2016). Examination of Berkovich indenter tip bluntness. Mater. Des..

[21] Fröhlich F., Grau P., Grellmann W. (1977). Performance and analysis of recording microhardness tests. Phys. Status Solidi.

[22] Attaf M.T. (2004). Connection between the loading curve models in elastoplastic indentation. Mater. Lett..

[23] Oliver W.C., Pharr G.M. (1992). An improved technique for determining hardness and elastic modulus using load and displacement sensing indentation experiments. J. Mater. Res..

[24] (2015). Metallic Material—Instrumented Indentation Test for Hardness and Materials Parameters—Part 1: Test Method.

[25] (2015). Metallic Materials—Instrumented Indentation Test for Hardness and Materials Parameters—Part 2: Verification and Calibration of Testing Machines.

[26] Sabzi H.E., Rivera-Díaz-del-Castillo P.E.J. (2020). Composition and process parameter dependence of yield strength in laser powder bed fusion alloys. Mater. Des..

[27] Kick F. (1885). Das Gesetz der Proportionalen Widerstände und Seine Anwendungen: Nebst Versuchen über das Verhalten Verschiedener Materialien Bei Gleichen.

[28] Suresh S., Nieh T.G., Choi B.W. (1999). Nano-indentation of copper thin films on silicon substrates. Scr. Mater..

[29] Bernhardt E.O. (1941). Über die Mikrohärte der Feststoffe im Grenzbereich des Kick’schen Ähnlichkeitssatzes. Int. J. Mater. Res..

[30] Marciniak Z., Duncan J.L., Hu J. (2002). Mechanics of Sheet Metal Forming.

[31] Wheeler J.M., Michler J. (2013). Elevated temperature, nano-mechanical testing in situ in the scanning electron microscope. Rev. Sci. Instrum..

[32] Benesty J., Chen J., Huang Y., Cohen I. (2009). Pearson Correlation Coefficient. Springer Top. Signal Process..

[33] Pitrmuc Z., Šimota J., Beránek L., Mikeš P., Andronov V., Sommer J., Holešovský F. (2022). Mechanical and Microstructural Anisotropy of Laser Powder Bed Fusion 316L Stainless Steel. Materials.

[34] Shamsujjoha M., Agnew S.R., Fitz-Gerald J.M., Moore W.R., Newman T.A. (2018). High Strength and Ductility of Additively Manufactured 316L Stainless Steel Explained. Metall. Mater. Trans. A Phys. Metall. Mater. Sci..

[35] Birnbaum A.J., Steuben J.C., Barrick E.J., Iliopoulos A.P., Michopoulos J.G. (2019). Intrinsic strain aging, Σ3 boundaries, and origins of cellular substructure in additively manufactured 316L. Addit. Manuf..

[36] Bertoli U.S., MacDonald B.E., Schoenung J.M. (2019). Stability of cellular microstructure in laser powder bed fusion of 316L stainless steel. Mater. Sci. Eng. A.

[37] Barkia B., Aubry P., Haghi-Ashtiani P., Auger T., Gosmain L., Schuster F., Maskrot H. (2020). On the origin of the high tensile strength and ductility of additively manufactured 316L stainless steel: Multiscale investigation. J. Mater. Sci. Technol..

[38] Li C., Liu Z.Y., Fang X.Y., Guo Y.B. (2018). Residual Stress in Metal Additive Manufacturing. Procedia CIRP.

[39] Casati R., Lemke J., Vedani M. (2016). Microstructure and Fracture Behavior of 316L Austenitic Stainless Steel Produced by Selective Laser Melting. J. Mater. Sci. Technol..

[40] Kok Y., Tan X., Wang P., Nai M., Loh N., Liu E., Tor S. (2018). Anisotropy and heterogeneity of microstructure and mechanical properties in metal additive manufacturing: A critical review. Mater. Des..

[41] Hitzler L., Hirsch J., Heine B., Merkel M., Hall W., Öchsner A. (2017). On the Anisotropic Mechanical Properties of Selective Laser-Melted Stainless Steel. Materials.

[42] Zeng Q., Gan K., Wang Y. (2021). Effect of Heat Treatment on Microstructures and Mechanical Behaviors of 316L Stainless Steels Synthesized by Selective Laser Melting. J. Mater. Eng. Perform..

[43] Mukherjee T., Zhang W., DebRoy T. (2017). An improved prediction of residual stresses and distortion in additive manufacturing. Comput. Mater. Sci..

[44] Jang J. (2009). Estimation of residual stress by instrumented indentation: A review. J. Ceram. Process. Res..

[45] Ulbricht A., Altenburg S.J., Sprengel M., Sommer K., Mohr G., Fritsch T., Mishurova T., Serrano-Munoz I., Evans A., Hofmann M. (2020). Separation of the Formation Mechanisms of Residual Stresses in LPBF 316L. Metals.

[46] Lavery N.P., Cherry J., Mehmood S., Davies H., Girling B., Sackett E., Brown S., Sienz J. (2017). Effects of hot isostatic pressing on the elastic modulus and tensile properties of 316L parts made by powder bed laser fusion. Mater. Sci. Eng. A.

[47] Motibane L.P., Tshabalala L.C., Mathe N.R., Hoosain S., Knutsen R.D. (2019). Effect of powder bed preheating on distortion and mechanical properties in high speed selective laser melting. IOP Conference Series: Materials Science and Engineering.

[48] Wang L., Felicelli S., Gooroochurn Y., Wang P.T., Horstemeyer M.F. (2008). Optimization of the LENS^®^ process for steady molten pool size. Mater. Sci. Eng. A.

[49] Qiu C., Al Kindi M., Aladawi A.S., Al Hatmi I. (2018). A comprehensive study on microstructure and tensile behaviour of a selectively laser melted stainless steel. Sci. Rep..

[50] Kurzynowski T., Gruber K., Stopyra W., Kuźnicka B., Chlebus E. (2018). Correlation between process parameters, microstructure and properties of 316 L stainless steel processed by selective laser melting. Mater. Sci. Eng. A.

[51] Wang X., Muñiz-Lerma J.A., Shandiz M.A., Sanchez-Mata O., Brochu M. (2019). Crystallographic-orientation-dependent tensile behaviours of stainless steel 316L fabricated by laser powder bed fusion. Mater. Sci. Eng. A.

[52] Raghavan N., Simunovic S., Dehoff R., Plotkowski A., Turner J., Kirka M., Babu S. (2017). Localized melt-scan strategy for site specific control of grain size and primary dendrite arm spacing in electron beam additive manufacturing. Acta Mater..

[53] Evangelou A., Stylianou R., Loizou A., Kim D., Liang A., Reed P., Constantinides G., Kyratsi T. (2023). Effects of process parameters and scan strategy on the microstructure and density of stainless steel 316 L produced via laser powder bed fusion. J. Alloys Metall. Syst..

[54] Song Y., Sun Q., Guo K., Wang X., Liu J., Sun J. (2020). Effect of scanning strategies on the microstructure and mechanical behavior of 316L stainless steel fabricated by selective laser melting. Mater. Sci. Eng. A.

[55] Hall E.O. (1951). The Deformation and Ageing of Mild Steel: III Discussion of Results. Proc. Phys. Soc. Sect. B.

[56] Petch N. (1953). The Cleavage Strength of Polycrystals. https://cir.nii.ac.jp/crid/1573387450368072704.

[57] Mukherjee M. (2019). Effect of build geometry and orientation on microstructure and properties of additively manufactured 316L stainless steel by laser metal deposition. Materialia.

[58] Bertsch K.M., de Bellefon G.M., Kuehl B., Thoma D.J. (2020). Origin of dislocation structures in an additively manufactured austenitic stainless steel 316L. Acta Mater..

[59] Jiang W.C., Gong J.M., Tu S.D., Chen H. (2009). Three-dimensional numerical simulation of brazed residual stress and its high-temperature redistribution for stainless steel plate-fin structure. Mater. Sci. Eng. A.

[60] Song Y., Yang J., Song K., Zhou Y., Huang T., Zhang C., Li T., Fan W. (2023). Effect of tensile stress annealing on residual stress and strength of C19400 alloy. J. Mater. Res. Technol..

[61] Zhao C., Bai Y., Zhang Y., Wang X., Xue J.M., Wang H. (2021). Influence of scanning strategy and building direction on microstructure and corrosion behaviour of selective laser melted 316L stainless steel. Mater. Des..

[62] Khodabakhshi F., Farshidianfar M., Gerlich A., Khajepour A., Trembošová V.N., Mohammadi M., Shakil S., Haghshenas M. (2023). Nanoindentation plasticity and loading rate sensitivity of laser additive manufactured functionally graded 316L and 410L stainless steels. Mater. Sci. Eng. A.

[63] Sames W.J., List F.A., Pannala S., Dehoff R.R., Babu S.S. (2016). The metallurgy and processing science of metal additive manufacturing. Int. Mater. Rev..

[64] Zhang Y., Allahkarami M., Hanan J.C. (2012). Measuring residual stress in ceramic zirconia–porcelain dental crowns by nanoindentation. J. Mech. Behav. Biomed. Mater..

[65] Serda M. (2016). Evaluation of Stacking Fault Energy in Anisotropic FCC Metal by Nanoindentation. Master’s Thesis.

[66] Mercelis P., Kruth J.P. (2006). Residual stresses in selective laser sintering and selective laser melting. Rapid Prototyp. J..

[67] Marattukalam J.J., Karlsson D., Pacheco V., Beran P., Wiklund U., Jansson U., Hjörvarsson B., Sahlberg M. (2020). The effect of laser scanning strategies on texture, mechanical properties, and site-specific grain orientation in selective laser melted 316L SS. Mater. Des..

[68] DebRoy T., Wei H.L., Zuback J.S., Mukherjee T., Elmer J.W., Milewski J.O., Beese A.M., Wilson-Heid A., De A., Zhang W. (2018). Additive manufacturing of metallic components—Process, structure and properties. Prog. Mater. Sci..

[69] Lu Y., Wu S., Gan Y., Huang T., Yang C., Junjie L., Lin J. (2015). Study on the microstructure, mechanical property and residual stress of SLM Inconel-718 alloy manufactured by differing island scanning strategy. Opt. Laser Technol..

[70] Fedorenko A., Fedulov B., Kuzminova Y., Evlashin S., Staroverov O., Tretyakov M., Lomakin E., Akhatov I. (2021). Anisotropy of Mechanical Properties and Residual Stress in Additively Manufactured 316L Specimens. Materials.

