# Supplementary material for: On the Mechanical Performance of an L-PBF 316l Part Using the Performance-Line Instrumented Indentation Test (PL-IIT)
Source: Materials (Basel). 2025 Mar 25;18(7):1462. doi: 10.3390/ma18071462 (PMC11989837; doi:10.3390/ma18071462)
Supplement: Supplementary file 1 [file materials-18-01462-s001.zip › materials-3488513-supplementary.pdf]

Supplementary material

Regression across the raw data and the average data was fitted for a_{85} (a), H_{IT} (b) and E_{IT} (c) across all five performance lines (PLs). The plots for the V line are shown in Figure 8 in the paper, and similar graphs for other D1, D2, H1 and H2 PLs are shown here in **Figure S1–S4** as supplementary material.

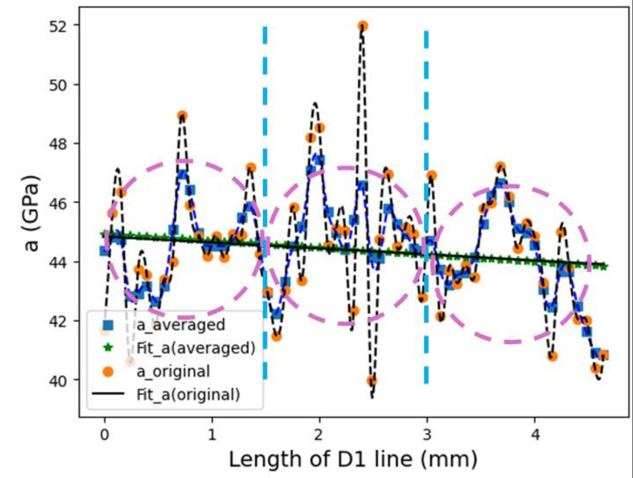

(a)

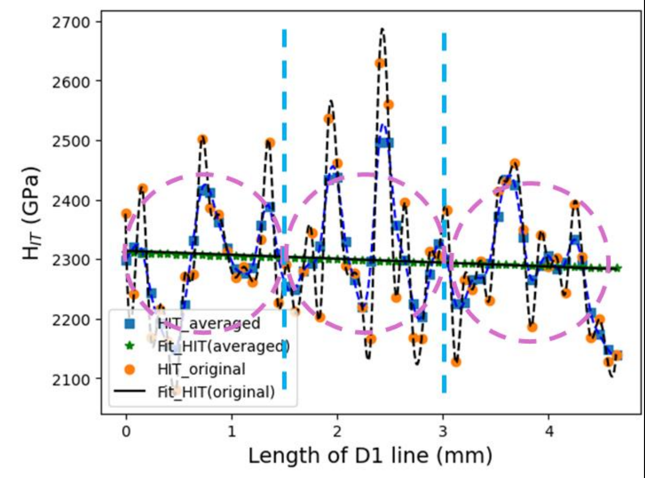

(b)

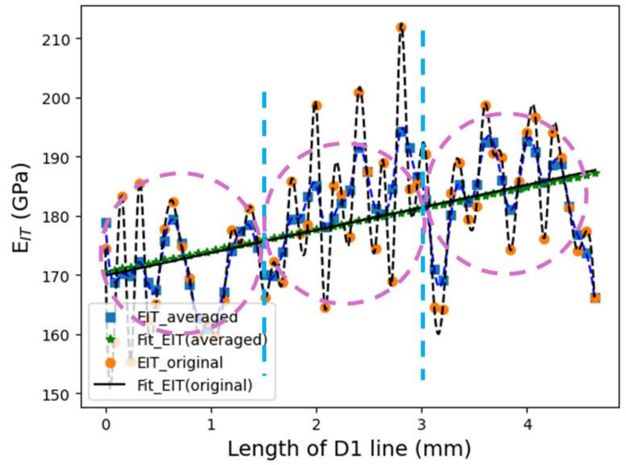

(c)

Figure S1. Regressions across the raw data (filled circles) and the average data (filled squares) for a_{85} (**a**), H_{IT} (**b**) and E_{IT} (**c**) along the D1 line. The black and blue dashed fitted lines relate to raw and averaged data points, respectively. The dashed circles delimit the segments partitioned over the D1 line with the corresponding average values.

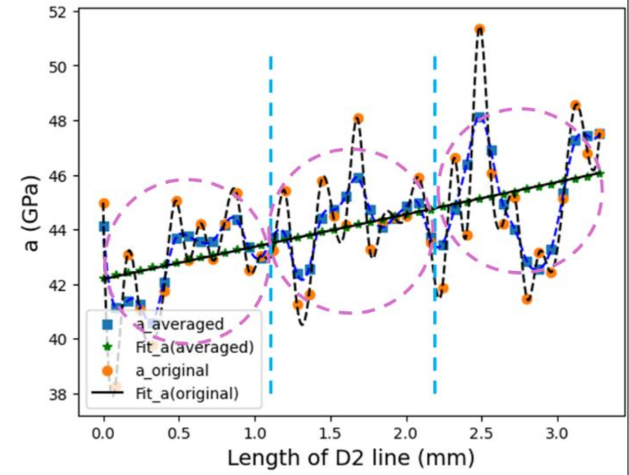

(a)

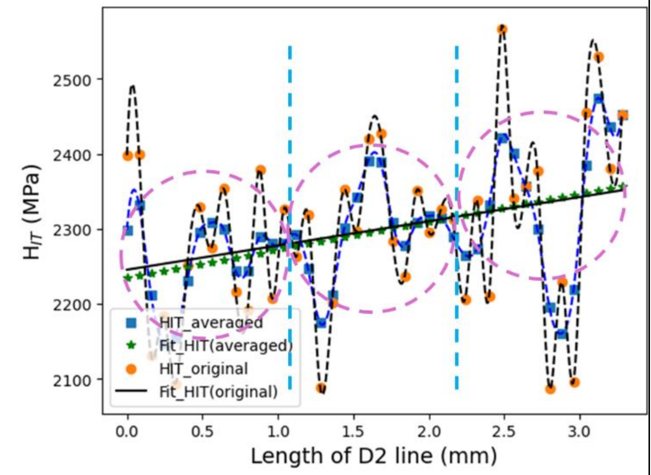

(b)

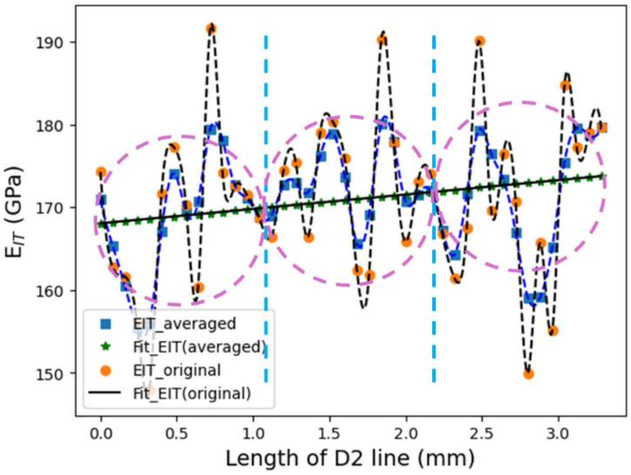

(b)

Figure S2. Regressions across the raw data (filled circles) and the average data (filled squares) for a_{85} (**a**), H_{IT} (**b**) and E_{IT} (**c**) along the D2 line.

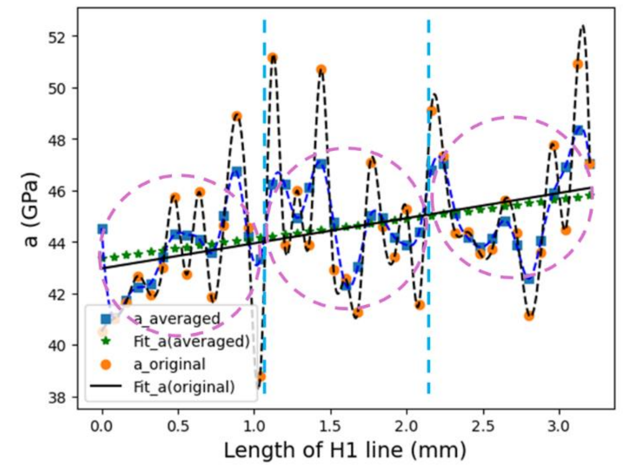

(a)

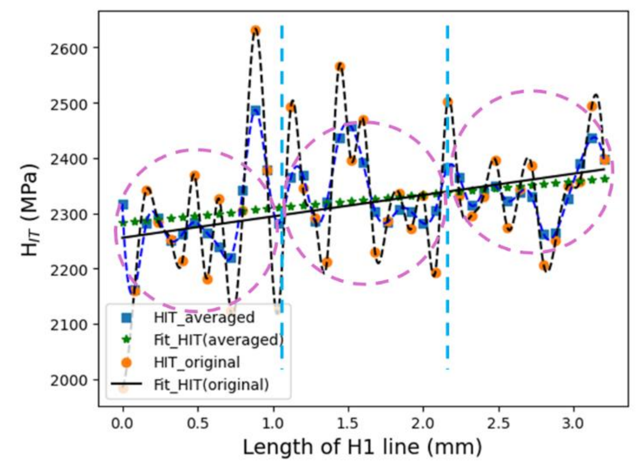

(b)

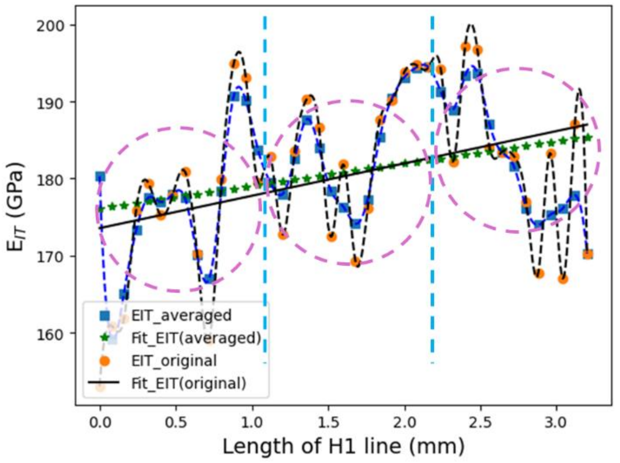

(c)

Figure S3. Regressions across the raw data (filled circles) and the average data (filled squares) for a_{85} (**a**), H_{IT} (**b**) and E_{IT} (**c**) along the H1 line.

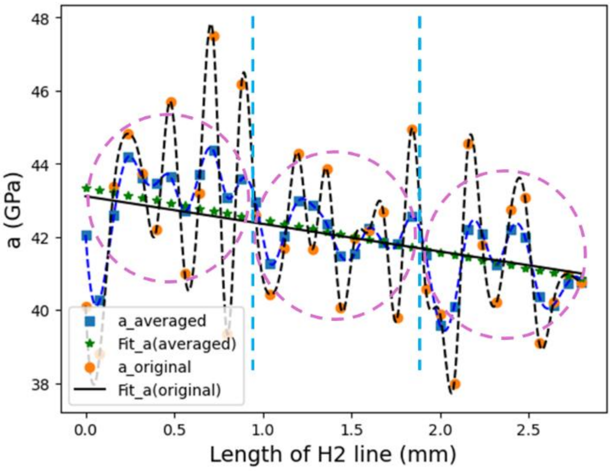

(a)

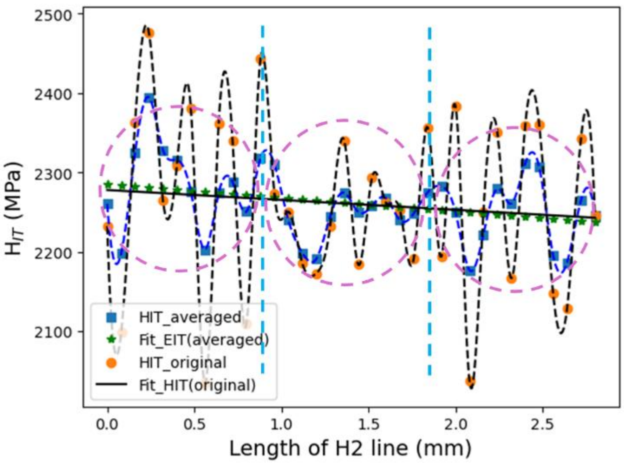

(b)

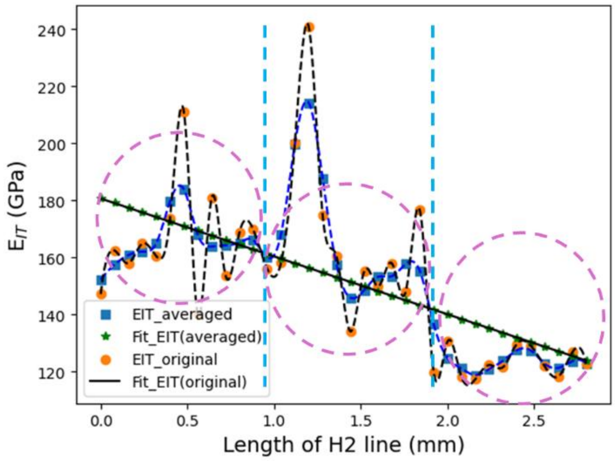

(c)

Figure S4. Regressions across the raw data (filled circles) and the average data (filled squares) for a_{85} (**a**), H_{IT} (**b**) and E_{IT} (**c**) along the H2 line.
